# Prevalence of Human Papillomavirus Genotypes among African Women with Normal Cervical Cytology and Neoplasia: A Systematic Review and Meta-Analysis

**DOI:** 10.1371/journal.pone.0122488

**Published:** 2015-04-14

**Authors:** Rebecca Kemunto Ogembo, Philimon Nyakauru Gona, Alaina J. Seymour, Henry Soo-Min Park, Paul A. Bain, Louise Maranda, Javier Gordon Ogembo

**Affiliations:** 1 Northeastern University, Boston, MA, United States of America; 2 University of Massachusetts Medical School, Worcester, MA, United States of America; 3 University of Massachusetts, Boston, MA, United States of America; 4 Yale University School of Medicine, New Haven, CT, United States of America; 5 Countway Library of Harvard Medical School, Boston, MA, United States of America; H. Lee Moffitt Cancer Center, UNITED STATES

## Abstract

**Background:**

Several meta-analyses confirmed the five most prevalent human papillomavirus (HPV) strains in women with and without cervical neoplastic diseases are HPV16, 18, 31, 52, and 58. HPV16/18 are the predominant oncogenic genotypes, causing approximately 70% of global cervical cancer cases. The vast majority of the women studied in previous analyses were from Europe, North America, Asia, and most recently Latin America and the Caribbean. Despite the high burden of cervical cancer morbidity and mortality in Africa, a robust meta-analysis of HPV genotype prevalence and distribution in African women is lacking.

**Methods and Findings:**

We systematically searched 14 major databases from inception to August 2013 without language restriction, following the Meta-Analysis of Observational Studies in Epidemiology and the Preferred Reporting Items for Systematic Reviews and Meta-Analyses guidelines. Seventy-one studies from 23 African countries were identified after screening 1162 citations and data abstracted and study quality appraised from 195 articles. HPV type-specific prevalence and distribution was estimated from 17,273 cases of women with normal cervical cytology; 1019 women with atypical squamous cells of undetermined significance (ASCUS); 1444 women with low-grade squamous intraepithelial lesion (LSIL); 1571 women with high-grade squamous intraepithelial lesion (HSIL); and 4,067 cases of invasive cervical carcinoma (ICC). Overall prevalence of HPV16/18 were 4.4% and 2.8% of women with normal cytology, 12.0% and 4.4% with ASCUS, 14.5% and 10.0% with LSIL, 31.2% and 13.9% with HSIL, and 49.7% and 18.0% with ICC, respectively. Study limitations include the lack of adequate data from Middle and Northern African regions, and variations in the HPV type-specific sensitivity of different genotyping protocols.

**Conclusions:**

To our knowledge, this study is the most comprehensive assessment of the overall prevalence and distribution of HPV genotypes in African women with and without different cervical neoplasias. We have established that HPV16/18 account for 67.7% of ICC cases among African women. Based on our findings, we highly recommend the administration of existing prophylactic vaccines to younger women not infected with HPV16/18 and an increase in HPV screening efforts for high-risk genotypes to prevent cervical cancer.

***Review registration*: **International Prospective Register of Systematic Reviews CRD42013006558.

## Introduction

Cervical cancer is the leading cause of mortality and years of life lost due to cancer in Africa [1] and predominantly affects women ages 15–44 years [2]. For decades, 12 highly carcinogenic human papillomavirus (HPV) genotypes have been recognized as the causative agent of cervical cancer [3,4]. Despite having the highest burden of risk factors associated with HPV infection, persistence, and progression to cervical cancer [5], comprehensive data on HPV genotype prevalence and distribution in Africa are lacking. The use of Papanicolaou (Pap) smear screening [6] and HPV prophylactic vaccines [7–9] are effective in preventing cervical cancer in most developed countries. However, their use in African countries remains very limited due to a variety of socio-economic and logistical barriers [10–13].

To date, several global meta-analyses on the distribution of HPV genotypes have confirmed the five most prevalent strains in women with normal cytology [14,15] and cervical neoplastic diseases [16–24] to be HPV16, 18, 31, 52, and 58. HPV16 and 18 were identified as the predominant oncogenic genotypes, causing approximately 70% of global cervical cancer cases [22], with the exception of women infected with HIV, in whom HPV58 is reported to be the second most dominant strain behind HPV16 [20]. The vast majority of the women studied in these investigations, however, were from Europe, North America [14,15,18–24], Asia [16], and most recently Latin America and the Caribbean [17]. Data on African women in these studies are highly variable and incomplete. Thus, the prevalence and distribution of HPV genotypes in Africa among women with normal cervical cytology, neoplastic lesions, and invasive cervical cancer (ICC) is required.

A comprehensive meta-analysis of HPV genotype prevalence and distribution among a large sample size of African women is needed to inform local, national, regional, and global policy to curb the spread of cervical cancer. In May 2013, the Global Alliance for Vaccine and Immunization (GAVI) announced the availability of HPV vaccines (Gardasil and Cervarix) for a reduced price of $4.50 per dose to low-income countries meeting the eligibility criteria [13,25]. With clear indications that the GAVI Alliance is committed to subsidizing HPV vaccines for low-income countries [13,26–29], many African nations are beginning to design effective strategies that address the potential challenges of vaccine delivery and screening for HPV and cervical cancer [13,28,29]. Epidemiological information on the prevalence and distribution of genital HPV infection in Africa is critical for planning for vaccine implementation; evaluating the possible impact of existing prophylactic HPV vaccines; and determining the relevant tools for HPV screening to prevent cervical cancer.

To our knowledge, this systematic review and meta-analysis of HPV genotype prevalence and distribution in Africa is the largest ever performed and includes 25,463 African women. The goal of this study was to establish the overall and type-specific prevalence and distribution of HPV genotypes among women with normal cervical cytology, neoplastic cervical lesions, and cancer in all five World Health Organization (WHO) regions of Africa.

## Methods

A systematic review protocol was performed adhering to the guidelines outlined in the Meta-Analysis of Observational Studies in Epidemiology (MOOSE) for systematic reviews [30] and the Preferred Reporting Items for Systematic Reviews and Meta-Analyses (PRISMA) statement for reporting systematic reviews and meta-analyses [31,32] (**See S1 Table**). The investigators wrote a protocol and registered it with the International Prospective Register of Systematic Reviews (identification number CRD42013006558) in November 2013 [33].

### Search Strategy

The search strategy was designed with a librarian (P.A.B.) to identify studies reporting HPV genotypes associated with cervical cancer or neoplasia in women living in Africa by performing a systematic search of literature databases from their earliest dates through August 27, 2013. The search, without any language restrictions, was performed in PubMed/MEDLINE (NCBI), Embase (Elsevier), Web of Science (Thomson), BIOSIS Preview (Thomson), Dissertations and Theses Full Text (ProQuest), Cochrane Central Register of Controlled Trials (Wiley), African Index Medicus (WHO), and POPLINE (K4Health). Exact strategies used to query each of these databases are reported in **S1 Appendix Detailed search strategy**. We also attempted to identify unpublished studies by examining ClinicalTrials.gov (NIH), International Clinical Trial Registry Platform (WHO), European Union Clinical Trials Register (EMA), and System for Information on Grey Literature in Europe (OpenGrey). The bibliographies of relevant reviews and eligible studies were also examined for additional sources. Databases containing conference proceedings or congress's annals, university theses, and experts were also consulted.

### Studies, eligibility criteria, and quality assessment

We used a comprehensive strategy to identify studies that included women living in African countries. Two reviewers (A.J.S. and J.G.O.) read the titles and abstracts to determine each study’s eligibility for full text review if (i) the article and relevant data were accessible, (ii) at least 10 women from an African country were included, (iii) cervical cytology/histology was confirmed by exfoliated cervical cells or fixed/fresh biopsy, (iv) HPV genotype-specific prevalence of at least three HPV genotypes was calculated, and (v) HPV genotype prevalence was calculated separately for each cervical lesion following the Bethesda classification [34,35]. There were no restrictions on the type of HPV assay method used. HPV genotypes were determined by molecular methods [signal amplified hybridization, polymerase chain reaction (PCR), DNA sequencing, type-specific probes, reverse line-blot hybridization, in situ hybridization, southern blot hybridization, or microarray] or by immunological techniques (including ELISA). Articles not meeting these five criteria were excluded. The reference lists of all the articles included in the study were also perused for additional information including non-English articles/abstracts.

Based on the aforementioned pre-defined criteria, two separate reviewers (A.J.S. and R.K.O.) independently reviewed full texts for data abstraction. When further clarification information was required, authors were contacted via email. Discrepancies were resolved by discussion or involvement of a third reviewer (J.G.O.). Two reviewers (A.J.S. and R.K.O.) independently assessed the methodological quality of studies and discrepancies were resolved by consensus [17]. Methodological quality assessment of observational studies was assessed by a checklist of essential items as outlined [24]. A list summarizing all 71 studies included for data abstraction and quality assessment is provided in **S2 Table**.

### Data items

If data or data subsets of the same population were published in multiple articles [36–38], only the publication with the largest sample size was used and the other reports were used as supplementary data. Two studies [39,40] had more detailed data in related doctoral theses, therefore the additional data from the theses were used to supplement the journal articles. Jones et al. [41] compared the results of HPV tests (clinician vs. self-administered), and data from the method considered to be the most sensitive was extracted. Four studies included data from women representing multiple countries [42–45]. Because the studies had clear lesion breakdowns, each of these articles was counted as an independent article in the analysis. Two studies [46,47] reported data from multiple countries without separating individual women studied by country. Though these papers had data broken down by lesion, at the regional level they were grouped into a mixed region category. Okolo et al. [48] independently analyzed for ICC, however, the data on normal cytology was duplicated in Thomas et al. [49]. Thus, the latter publication was used as the source of data for abstraction.

### Data extraction

The key information retrieved for analysis included: participant characteristics (population, mean or median age, standard deviation of age), study characteristics (study design, period of data collection, sample size), laboratory location where the samples were processed and genotyped, geographical settings (region according to WHO classification), country, distribution of cases by histological type, tissue source, PCR primers (GP5+/6+, MY09/11, PGMY09/11, SPF10 or individual laboratory designed primers), detailed HPV detection and genotyping methodology, number of HPV positive women, and overall HPV prevalence where given. Data was extracted for both high risk (HR) and low-risk (LR) HPV genotypes [50]. Studies reporting multiple infections with any HPV genotype were also extracted. Specimens negative for beta-globin were not included in the sample size. Cases were classified into five grades of cervical diagnosis by cytology and/or by histology as previously outlined [34,35]. This included women with: 1) normal cervical cytology; 2) atypical squamous cells of undetermined significance (ASCUS); 3) low-grade intraepithelial lesion (LSIL), including cervical intraepithelial neoplasia grade 1 (CIN1); 4) high-grade squamous intraepithelial lesion (HSIL), including CIN2-3; and 5) ICC [including squamous cell carcinoma (SCC) and adeno/adenosquamous carcinoma].

### Statistical analysis

Overall HPV prevalence was defined as the proportion of tested individuals that were positive for any HPV infection expressed as a percentage [(the number of HPV positive women/the total number tested)*100]. Similarly, HPV type-specific prevalence by cytology or histology was also calculated for those testing positive for the specific HPV type by cytological category according to the 2001 Bethesda System [34,35]. HPV type-specific prevalence was defined as the proportion of women testing positive for a specific HPV genotype among all HPV positive women tested for that genotype. In HPV type–specific prevalence, only studies that tested for a particular HPV type contributed to the analysis for that type, so sample sizes differed between the type-specific analyses. Multiple HPV infections were separated into constituent types, thus type-specific prevalence represents both single and multiple infections as previously defined [17].

Statistical analysis was conducted as described by Ciapponi et al. [17], using StatsDirect meta-analysis software (Cheshire, United Kingdom) and STATA 13.0 (College Station, TX). Briefly, the sample size (N), number of cases for HPV type (rows), and each cytology/histology classification (column) were calculated by summing the number of all study participants and the number of events in each row/column combination. Raw prevalence was computed by dividing the number of events by sample size, N. Exact 95% confidence interval was computed under binomial. Studies in which the HPV-type was not assessed or was not reported were excluded from the HPV type-specific analyses. Studies in which age was not reported were recorded and analyzed as missing age.

The computed proportions were arcsine square root transformed using the Freeman-Tukey method to stabilize the variance [51]. The meta-analysis was then carried out on the transformed proportions, with the inverse of the variance of the transformed proportion serving as study-specific weight. The arcsine transformations were done to stabilize the variance of simple proportions [17]. We further applied DerSimonian-Laird weights for the random effects model [52] when heterogeneity between studies included in the analysis was found. The I-squared (I^2^) statistic was used to quantify the heterogeneity between studies.

Additionally, a meta-regression using grouped logistic regression analysis adjusted for age-group was performed to further identify the possible sources of heterogeneity. We then computed lesion-specific age-adjusted prevalence for any HPV and HPV16 for each region. We then used LSMEANS statement with the ILINK options in the SAS LOGISTIC procedure to back transform log-odds into proportions and generate 95% confidence intervals.

#### Ethical statement

No institutional review board approval was required for this study.

## Results

### Selection of studies for review

**Fig 1:**
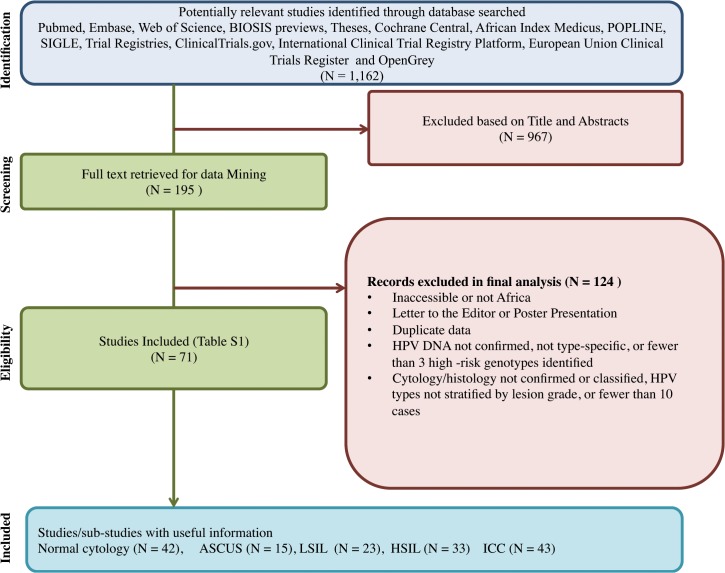
Study flow diagram. Abbreviation: ASCUS: Atypical squamous cells of undetermined significance; LSIL: Low-grade squamous intraepithelial lesions; HSIL: High-grade squamous intraepithelial lesions; ICC: Invasive cervical cancer (this includes SCC and ADC).

A total of 1162 citations were retrieved and 195 full articles were reviewed. Data was abstracted from 71 epidemiological studies with individual-level data meeting the inclusion criteria. The study flow diagram is shown in **Fig 1**.

### Study characteristics

Data were collected from studies conducted between 1968 [53] to 2010 [44]. With the exception of most studies from South Africa [41,44,54–68], a study from Morocco [69], Ethiopia and Sudan [42], all HPV genotyping of African participants were performed in laboratories in Europe (France, Spain, Great Britain, Italy, Sweden, the Netherlands, Belgium and Denmark), Japan, or the United States of America (**S4 Table**). Fifty-six studies reported multiple infections with any HPV type-specific. A total of 30,444 women from 23 African countries were recruited to participate in the various studies included in the systematic review. Beta globin tests were done on 27,915 sample specimens. Finally, HPV DNA testing was done on 25,463 sample specimens from women who had a positive beta globin test result. Their distribution by cytological type and geographical region is given in **Table 1**.

**Table 1:** Regional and country specific distribution of studies, study size and prevalence of HPV DNA by cervical disease grade and region.

African Region (No. studies)	Country [Reference]	Total (studies = 71)		Normal Cytology (studies = 42)		ASCUS (studies = 15)		LSIL (studies = 23)		HSIL (studies = 33)		ICC (studies = 43)	
		Tested, N	HPV (+), n	Tested, N	HPV+, n (%)	Tested, N	HPV+, n (%)	Tested, N	HPV+, n(%)	Tested, N	HPV+, n(%)	Tested, N	HPV+, n(%)
Eastern (26)	Ethiopia [42, 70, 71], Kenya [39, 72–79], Mozambique [36, 80, 81], Rwanda [82], Tanzania [43, 83–85]^**Ŧ**^, Uganda [43, 53, 86–88] ^**Ŧ**^, Zambia [40, 89], Zimbabwe [90–92]	10246	5146	6640	1907 (29)	440	260 (59)	605	417 (68)	657	563 (85)	1904	1725 (90)
Middle (3)	Equatorial Guinea [93], Zaire (DRC) [94]	30	23	.	.	.	.	8	6 (60)	19	14 (74)	3	3 (100)
Northern (6)	Algeria [37, 38, 43] ^**Ŧ**^, Morocco [69, 95, 96], Sudan [42]	2510	725	1865	219 (12)	46	4 (7)	24	4 (17)	8	4 (50)	517	473 (90)
Southern (19)	Botswana [97, 98], South Africa [41, 44, 54–68] **	4983	3392	3030	1505 (50)	207	126 (61)	521	448 (87)	768	645 (85)	457	388 (85)
Western (16)	Benin [43, 99] ^**Ŧ**^, Cote d’Ivoire [100–102], Gambia [103], Ghana [44] **, Guinea [43, 104] ^**Ŧ**^, Mali [43, 45, 105] * ^**Ŧ**^, Nigeria [44, 48, 49, 106] **, Senegal [45, 107–109]*,	7265	2878	5600	1363 (24)	326	85 (26)	286	191 (67)	102	87 (85)	951	845 (89)
Mixed studies	Tanzania & South Africa [47], Kenya & South Africa [46],	429	334	138	58 (42)	.	.	.	.	17	16 (94)	235	225 (96)
Overall (71)	.	**25463**	**12498**	**17273**	**5074 (29)**	**1019**	**474 (47)**	**1444**	**1087 (74)**	**1571**	**1371 (85)**	**4067**	**3741 (89)**

Abbreviations:

ASCUS: Atypical squamous cells of undetermined significance; LSIL: Low-grade squamous intraepithelial lesions; HSIL: High-grade squamous intraepithelial lesions; ICC: Invasive cervical cancer included both SCC and ADC.

“Mixed studies” refers to study including women from more than one country without separating individual women to the country where they came from.

*Senegal & Mali counted here as two sub-studies from [45];

** Studies from Ghana, Nigeria, South Africa were from one study [44], but counted here as a sub-study each;

^Ŧ^ All these countries (Algeria, Benin, Guinea, Mali, Uganda, Tanzania) were in one study [43], counted here as stand-alone sub-studies.

Out of 24,643 women tested for any HPV infection, 17,273 had normal cytology; 1,019 were classified as ASCUS; 1,444 were diagnosed with LSIL; 1,571 were HSIL-positive; and 4,067 had ICC. In addition, a total of 346 women were reported as HSIL or higher (either HSIL or ICC), 505 were classified as abnormal without separation into a specific lesion grade, and 257 were of an unspecified low lesion type. The last three categories were expunged from further analysis.

Based on the WHO classification of geographic regions of Africa, Eastern Africa had the highest number of women included in the study (10,246), followed by Western Africa (7,265), Southern Africa (4,983), and Northern Africa (2,510) (**Table 1**). Middle Africa was the least represented region, including only 30 women from two studies (Equatorial Guinea and Democratic Republic of Congo, formerly Zaire). South Africa (4,962) contributed the largest study size followed by Tanzania (4,398), Senegal (2,360), Nigeria (2,289) and Kenya (2,034). Morocco, Cote d’Ivoire, and Algeria each contributed over 1,000 women to the study. Each of the remaining 15 countries had fewer than 1,000 women included in the study. Two studies [46,47] included 429 women from three different countries without identifying the subjects to their specific country of origin.

### Meta-analysis results according to Bethesda Classification

The meta-analysis of HPV type-specific prevalence by lesion types according to the Bethesda classification of women with normal cervical cytology and cervical neoplastic lesions (ASCUS, LSIL, HSIL and ICC) are presented in **Table 2**. Further sub-group analysis of the 71 studies was performed based on source of tissues, country and Gross National Income (GNI) classification according to the World Bank **Table 3**and **S3 Table**. There were no statistically significant differences in all the sub-group analysis for any HPV in women with normal cervix, neoplasia or ICC. Age-adjusted prevalence for any HPV and HPV16 categorized by region and lesion type is provided in **Table 4**.

**Table 2:** Prevalence of HPV by Bethesda Classification by cytology.

**HPV types**		Normal			ASCUS			LSIL			HSIL			ICC		
HIGH-RISK	Type	Sample Size, N	# Cases (studies)	**Prevalence (%) (95%CI)**	Sample Size, N	# Cases (studies)	**Prevalence (%) (95%CI)**	Sample Size, N	# Cases (studies)	**Prevalence (%) (95%CI)**	Sample Size, N	# Cases (studies)	**Prevalence (%)(95%CI)**	Sample Size, N	# Cases (studies)	**Prevalence (%) (95%CI)**
	hpv16	17273	766 (42)	4.4 (4.1–4.7)	1019	122 (15)	12 (10.0–14.1)	1444	213 (23)	14.5 (12.8–16.4)	1571	504 (33)	31.2 (28.9–33.5)	4067	2078 (43)	49.7 (48.2–51.2)
	hpv18	17273	491 (42)	2.8 (2.6–3.1)	1019	45 (15)	4.4 (3.2–5.9)	1444	146 (23)	10.0 (8.5–11.6)	1571	224 (33)	13.9 (12.2–15.6)	4067	751 (43)	18.0 (16.8–19.2)
	hpv31	17273	329 (42)	1.9 (1.7–2.1)	1019	27 (15)	2.6 (1.8–3.8)	1444	67 (23)	4.6 (3.6–5.8)	1571	133 (33)	8.2 (6.9–9.7)	3845	104 (38)	2.7 (2.2–3.3)
	hpv33	17273	273 (42)	1.6 (1.4–1.8)	1019	24 (15)	2.4 (1.5–3.5)	1444	73 (23)	5.0 (3.9–6.2)	1571	167 (33)	10.3 (8.9–11.9)	3540	167 (32)	4.7 (4–5.5)
	hpv35	17105	511 (40)	3.0 (2.7–3.3)	1019	40 (15)	3.9 (2.8–5.3)	1444	164 (23)	11.2 (9.6–12.9)	1571	216 (33)	13.4 (11.7–15.1)	3619	198 (31)	5.5 (4.8–6.3)
	hpv39	16128	205 (38)	1.3 (1.1–1.5)	964	28 (13)	2.9 (1.9–4.2)	1386	50 (20)	3.6 (2.7–4.7)	1510	55 (31)	3.4 (2.6–4.4)	3605	39 (36)	1.1 (0.8–1.5)
	hpv45	17008	339 (40)	2.0 (1.8–2.2)	1010	37 (14)	3.7 (2.6–5.0)	1444	76 (22)	5.2 (4.1–6.5)	1445	104 (31)	6.4 (5.3–7.7)	4067	408 (42)	10.0 (9.1–10.9)
	hpv51	17105	363 (40)	2.1 (1.9–2.3)	1019	31 (15)	3 (2.1–4.3)	1444	84 (23)	5.7 (4.6–7.0)	1571	92 (33)	5.7 (4.6–6.9)	3619	76 (32)	2.1 (1.7–2.6)
	hpv52	16128	518 (38)	3.2 (2.9–3.5)	964	66 (13)	6.8 (5.3–8.6)	1428	148 (21)	10.4 (8.8–12.1)	1438	191 (30)	11.8 (10.3–13.5)	3427	146 (30)	4.3 (3.6–5)
	hpv56	16128	253 (38)	1.6 (1.4–1.8)	964	22 (13)	2.3 (1.4–3.4)	1386	89 (20)	6.4 (5.2–7.8)	1415	72 (29)	4.5 (3.5–5.6)	3427	33 (30)	1.0 (0.7–1.3)
	hpv58	16128	421 (38)	2.6 (2.4–2.9)	964	26 (13)	2.7 (1.8–3.9)	1428	89 (21)	6.2 (5.0–7.6)	1438	183 (30)	11.3 (9.8–13)	3427	83 (30)	2.4 (1.9–3)
	hpv59	16128	192 (40)	1.2 (1–1.4)	964	19 (13)	2 (1.2–3.1)	1386	54 (20)	3.9 (2.9–5.1)	1415	35 (29)	2.2 (1.5–3)	3618	27 (36)	0.7 (0.5–1.1)
LOW-RISK	hpv6	17273	132 (42)	0.8 (0.6–0.9)	1019	12 (15)	1.2 (0.6–2.0)	1444	38 (23)	2.6 (1.8–3.5)	1571	39 (33)	2.4 (1.7–3.3)	4181	42 (43)	1 (0.7–1.4)
	hpv11	13680	59 (36)	0.4 (0.3–0.6)	964	5 (13)	0.5 (0.2–1.2)	1230	17 (20)	1.4 (0.8–2.2)	1336	18 (29)	1.1 (0.7–1.8)	2919	12 (31)	0.4 (0.2–0.7)
	hpv53	14664	258 (36)	1.8 (1.6–2.0)	964	28 (13)	2.9 (1.9–4.2)	1188	85 (19)	7.2 (5.8–8.8)	1232	92 (28)	5.6 (4.6–6.9)	3129	19 (28)	0.6 (0.4–0.9)
	hpv54	13119	99 (31)	0.8 (0.6–0.9)	964	25 (13)	2.6 (1.7–3.8)	981	30 (17)	3.1 (2.1–4.3)	1085	34 (25)	2.1 (1.5–2.9)	2233	1 (20)	0 (0–0.2)
	hpv66	14797	201 (36)	1.4 (1.2–1.6)	964	28 (13)	2.9 (1.9–4.2)	1188	60 (19)	5.1 (3.9–6.5)	1232	86 (28)	5.3 (4.3–6.5)	3209	23 (27)	0.7 (0.5–1.1)
	hpv68	15819	245 (37)	1.5 (1.4–1.8)	964	19 (13)	2 (1.2–3.1)	1428	50 (21)	3.5 (2.6–4.6)	1438	62 (30)	3.8 (3.0–4.9)	2051	30 (21)	1.5 (1–2.1)
	hpv70	13098	108 (30)	0.8 (0.7–1)	885	9 (12)	1 (0.5–1.9)	1002	31 (18)	3.1 (2.1–4.4)	833	22 (22)	1.4 (0.9–2.1)	2525	5 (27)	0.2 (0.1–0.5)
	hpv82	11558	20 (27)	0.2 (0.1–0.3)	964	2 (13)	0.2 (0–0.7)	821	11 (16)	1.3 (0.7–2.4)	1013	9 (24)	0.6 (0.3–1.1)	1485	0 (21)	0 (0–0.2)
	hpvmult*	15157	1399 (37)	9.2 (8.8–9.7)	897	474 (14)	20.6 (18–23.4)	1054	475(20)	44.6 (41.6–47.7)	1519	1371 (29)	38.5 (36.1–40.9)	3553	545 (33)	15.3 (14.2–16.6)
	anyhpv	17273	5074 (42)	29.3 (28.6–30.0)	1019	185 (15)	46.5 (43.4–49.6)	1444	1087(23)	74.2 (71.9–76.4)	1571	622 (33)	84.8 (82.9–86.5)	4067	3741 (43)	89.5 (88.5–90.4)

hpvmult*—Multiple HPV genotypes.

**Table 3:** Overall prevalence of any HPV according to year of publication, primers, and age categories.< /Table_Caption>

		**ICC**		**HSIL**		**LSIL**		**ASCUS**		**Normal**	
		**Cases (Studies)**	**% Prevalence (95% CI)**	**Cases (Studies)**	**% Prevalence (95% CI)**	**Cases (Studies)**	**% Prevalence (95% CI)**	**Cases (Studies)**	**% Prevalence (95% CI)**	**Cases (Studies)**	**% Prevalence (95% CI)**
YEAR OF PUBLICATION	1986–1990	13 (1)	100 (87.6–112.4)	98 (1)	67.4 (57.7–77.0)						
	1991–1995	266 (8)	85.4 (77.3–93.6)	74(2)	70.8 (22.5–119.1)	6 (1)	75.0 (44.1–106)			15 (1)	19.5 (10.1–28.9)
	1996–2000	181 (3)	76.4 (62.0–90.8)	60 (1)	81.7 (71.2–92.1)	144 (3)	72.9 (50.3–95.7)	2 (1)	22.2(-6.4–50.8)	228 (6)	21.9 (14.8–29.2)
	2001–2005	646 (8)	98.4 (97.2–99.6)	426 (6)	88.6 (81.1–95.8)	72 (3)	70.2 (39.6–100.9)	123 (2)	55.1 (-12.9–123.1)	818 (8)	26.5 (18.0–35.0)
	2006–2010	1060 (11)	91.2 (86.1–96.2)	335 (13)	93.4 (88.8–98.0)	389 (10)	81.6 (71.1–92.1)	186 (172)	78.4 (52.3–104.5)	1672 (15)	59.1 (46.5–71.7)
	2011–2015	1575 (13)	88.6 (85.2–91.9)	624 (11)	88.8 (83.8–93.8)	476 (8)	72.8 (60.3–85.3)	172 (4)	47.9 (39.4–56.5)	2341 (13)	35.9 (23.2–48.7)
	**Total**	**3741 (42)**	**90.5 (88.2–92.7)**	**1617 (33)**	**87.2 (83.4–91.1)**	**1087 (23)**	**75.8 (68.3–83.4)**	**483 (15)**	**65.7 (48.0–83.4)**	**5074 (43)**	**40.2 (33.9–46.5)**
PRIMER	(GP)5+/6+	1152 (10)	93.4 (90.3–96.4)	159 (5)	80.5 (67.2–93.6)	193 (3)	58.4 (45.4–71.4)	164 (3)	61.9 (31.5–92.2)	1054 (12)	34.7 (22.9–46.7)
	In-house primer	385 (8)	86.3 (77.5–95.1)	238 (4)	75.6 (57.5–93.8)	41 (2)	70.2 (18.9–121.5)	2 (1)	22.2 (-6.4–50.8)	88 (4)	24.7 (10.2–39.1)
	PGMY&GP	.	.	.	.	.	.			183 (1)	40.3 (35.7–44.9)
	PGMY09/11	351 (11)	90.6 (85.2–96.0)	569 (15)	92.7 (88.7–96.6)	523 (11)	86.9 (79.9–93.9)	149 (7)	85.6 (66.1–105.0)	1773 (13)	60.3 (44.4–76.2)
	SPF	1772 (13)	89.8 (85.5–94.2)	180 (5)	94.2 (90.4–98.0)	77 (3)	79.8 (59.3–100.2)	70 (1)	41.4 (33.8–49.1)	922 (5)	40.3 (18.9–61.*)
	MY09/11	81 (2)	75.6 (38.1–113.2)	225 (5)	82.0 (70.4–93.5)	253 (6)	66.3 (46.5–86.1)	89 (3)	34.2 (3.7–64.6)	1054 (8)	24.7 (15.9–33.5)
	**Total**	**3741 (42)**	**90.5 (88.2–92.7)**	**1379 (33)**	**87.2 (83.3–91.1)**	**1087 (23)**	**75.8 (68.3–83.4)**	**474 (15)**	**63.5 (45.3–81.6)**	**5074 (42)**	**40.2 (33.9–46.5)**
AGE CATEGORY	15–24	.	.	9 (1)	100.0 (83.2–116.8)	10 (1)	100.0 (84.6–115.4)			217 (2)	48.2 (14.7–81.6)
	25–34	599 (11)	92.5 (86.3–98.7)	390 (11)	86.7(78.5–95.0)	515 (8)	81.5 (69.7–93.4)	147 (6)	80.9 (61.9–99.9)	886 (11)	50.5 (37.1–63.8)
	35–44	723 (9)	94.7 (90.9–98.5)	835 (14)	91.3 (87.8–94.8)	529 (13)	64.8 (52.4–77.2)	301 (7)	51.7 (27.0–76.4)	2579 (20)	36.1 (26.9–45.2)
	45–54	1627 (16)	91.7 (88.8–94.6)	12 (2)	87.1 (67.3–107.0)	11 (1)	100.0 (85.8–114.2)	3 (1)	6.5 (-1.7–14.8)	1043 (6)	31.6 (14.9–48.3)
	55–64	480 (5)	88.1 (82.2–94.0)	.	.					.	.
	>65	.	.	.	.					.	.
	Missing	312 (3)	72.6 (52.2–93.1)	133 (6)	80.4 (62.9–97.9)	22 (2)	91.9 (69.0–114.8)	23 (1)	100.0 (92.6–107.4)	349 (4)	41.4 (38.0–44.8)
	**Total**	**3741 (44)**	**90.5 (88.2–92.7)**	**1379 (34)**	**87.2 (83.3–91.1)**	**1087 (25)**	**75.8 (68.3–83.4)**	**474 (15)**	**63.5 (45.3–81.6)**	**5074 (43)**	**40.2 (33.9–46.5)**

**Abbreviations**: Lesions-ASCUS: Atypical squamous cells of undetermined significance; LSIL: Low-grade squamous intraepithelial lesions; HSIL: High-grade squamous intraepithelial lesions; ICC: Invasive cervical cancer (this included SCC and ADC). CI: Confidence interval; N: Number of cases tested for a given HPV type. Blank boxes means indeterminate.

Heterogeneity was found to be substantial, ICC: I^2^ = 88.8%, 43 studies; HSIL I^2^ = 81.5%, 33 studies; LSIL I^2^ = 93.3%, 23 studies; ASCUS I^2^ = 98.5%, 15 studies; and Normal I^2^ = 99.1%, 42 studies, all p-values <0.001. Random effects age-group adjusted meta-analysis using logistic regression analysis were performed for every HPV genotype.

**Table 4:** Lesion specific age-adjusted prevalence for any HPV and HPV16 for each region.

	**Age**	**Any HPV**	**HPV16**	**Region**	**Any HPV**	**HPV16**
Normal	<25	36.0(32.3–39.9)	6.8(5.1–9.1)	Eastern	33.1(31.6–34.5)	4.5(4.0–5.3)
	25-<35	47.7(45.4–50.0)	5.9(5.0–7.1)	Southern	58.5(56.2–60.7)	11.2(9.6–13.1)
	35-<45	26.8(26.2–27.6)	4.1(3.7–4.5)	Western	28.7(27.1–30.8)	4.2(3.5–5.0)
	45-<55	25.3(23.8–26.9)	3.7(3.2–4.5)	Middle	-	-
	= >55’	-	-	Northern	16.9(14.9–19.2)	3.0(2.2–4.2)
ASCUS	<25	-	-	Eastern	60.2(55.4–64.8)	15.6(11.8–19.7)
	25-<35	65.0(58.6–71.0)	6.6(4.0–10.7)	Southern	62.7(55.7–69.2)	6.3(3.8–10.1)
	35-<45	41.2(37.9)	13.5(11.3–16.1)	Western	29.6(23.8–36.3)	1.7(0.9–3.3)
	45-<55	-	-	Middle	-	-
	= >55’	-	-	Northern	-	-
LSIL	<25	100(0–100)	10.0(2.5–32.4)	Eastern	-	-
	25-<35	76.0(72.6–79.0)	16.8(14.2–19.8)	Southern	-	-
	35-<45	70.8(67.4–73.9)	10.8(8.7–13.2)	Western	-	-
	45-<55	100(0–100)	100(0–100)	Middle	-	-
	= >55’	-	-	Northern	-	-
HSIL	<25	100.0(0–100)	5.6(0.7–3.1)	Eastern	-	27.2(17.3–40.0)
	25-<35	81.5(77.7–84.7)	28.6(24.7–32.8)	Southern	-	28.9(18.2–42.7)
	35-<45	80.0(73.1–85.4)	31.8(29.1–34.7)	Western	-	26.2(14.5–42.7)
	45-<55	85.6(57.2–96.4)	64.3(37.6–84.3	Middle	-	-
	= >55’	-	-	Northern	-	-
ICC	<25	-	-	Eastern	91.4(89.9–92.9)	-
	25-<35	91.7(89.4–93.6)	47.5(43.7–51.3)	Southern	82.1(77.3–86.0)	-
	35-<45	88.2(86.2–90.0)	47.2(44.3–50.2)	Western	87.3(84.4–89.7)	-
	45-<55	91.1(89.7–92.4)	52.8(50.4–55.1)	Middle	-	
	= >55’	-	49.9(45.7–54.1)	Northern	89.0(85.2–91.9)	-

'-' Age-group adjusted prevalence cannot be estimated because of sparse data.

### Prevalence of HPV genotypes in women with normal cervical cytology

Systematic review included 42 studies testing for cervical HPV infection in 17,273 women with normal cytology. This is the highest number of African women with normal cervical cytology ever included in a single HPV prevalence meta-analysis study. Most of the women tested for HPV infections were from Eastern Africa (n = 6,640), followed by Western Africa (n = 5,600), Southern Africa (n = 3,030) and Northern Africa (n = 1,865). A total of 138 individuals were from studies that contained mixed countries (**Table 1**). HPV16, 52, 35, 18, 58, 51, 45, 31, 53 and 56 were the ten most common genotypes in women with normal cervical cytology in descending order **(Table 2**).

**Fig 2:**
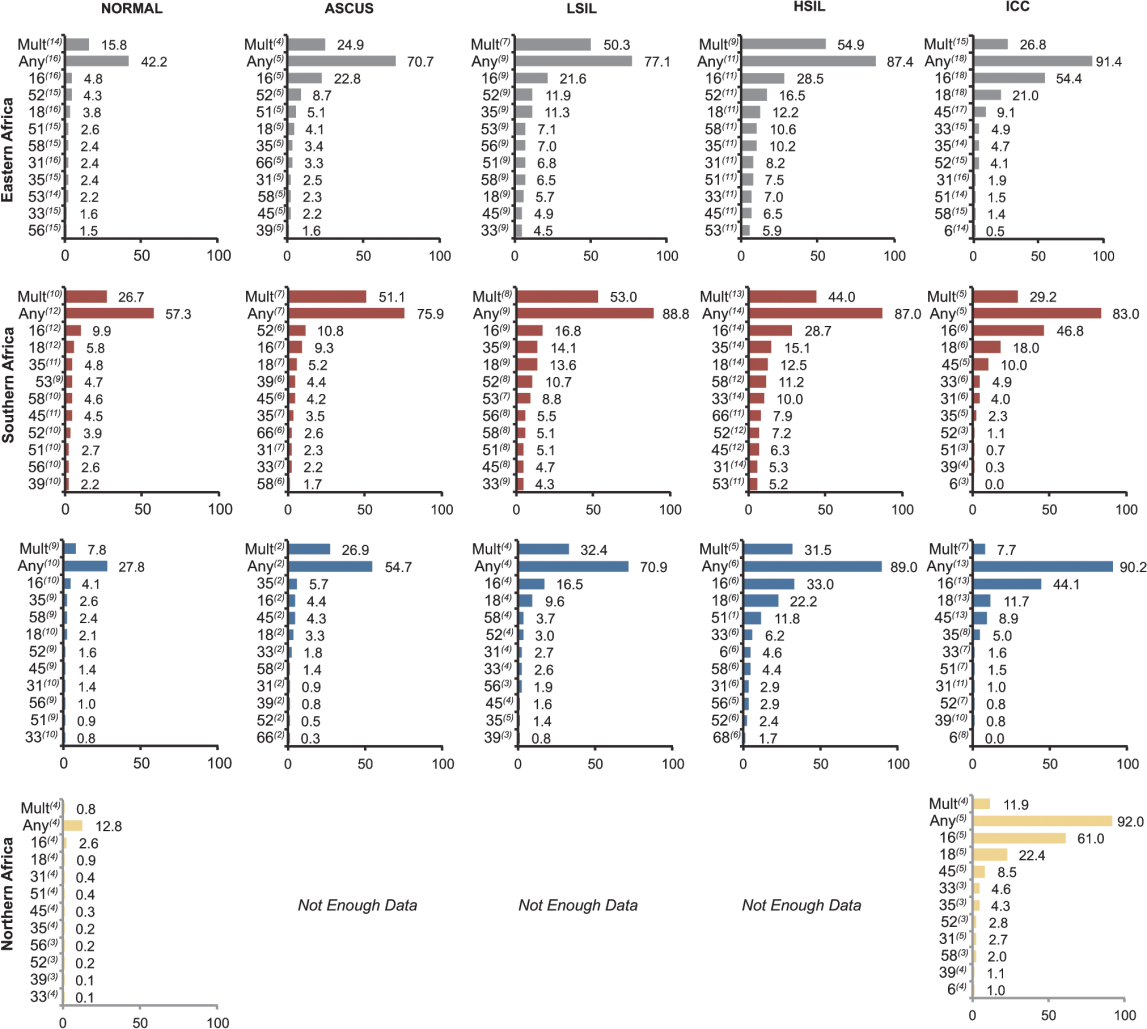
The ten most common HPV genotypes by regions and lesion types according to Bethesda classifications, respectively. Type specific prevalence is weighted by study size. The superscript numbers in brackets represent the number of studies included for the specific HPV genotype. There was not enough data from the middle African region.

There was wide variation in any HPV infection rates based on region, with Southern Africa (57.3%) having the highest prevalence, followed by Eastern Africa (42.2%), Western Africa (27.8%), and Northern Africa (12.8%) (**Fig 2**left panel). Type-specific HPV16 and 18 were higher among women with normal cervical cytology from Southern Africa compared to other regions at 9.9% and 5.8% respectively (**Fig 2**). No study representing women from Middle Africa met the inclusion criteria.

Overall, 39 studies stratified eligible women screened for HPV infection according to age groups. Analysis of these studies revealed that the age group 25–34 years had the highest overall HPV prevalence at 50.5% (95% CI: 37.1–63.8; I^2^ = 97.2%), closely followed by the age group 15–24 years with 48.2% (95% CI: 14.7–81.6; I^2^ = 96.6%). Age groups 35–44 and 45–54 years had HPV prevalence of 36.1% (95% CI: 26.9–45.2; I^2^ = 99.3%) and 31.6% (95% CI: 14.9–48.3; I^2^ = 99.2%), respectively, all p-values <0.001 (**Table 3**). Further lesion-specific age-adjusted prevalence for any HPV and HPV16 for each region is provided in **Table 4**.

### Prevalence of HPV genotypes in Women with ASCUS and LSIL

To date, only one meta-analysis of HPV type-specific prevalence among women from Africa with ASCUS and LSIL exists [21]. However, in that study there were less than 300 women included in each lesion type. Our meta-analysis included fifteen studies testing for HPV infection in 1,019 women diagnosed with ASCUS. Most of the women tested for HPV infections were from Eastern Africa (n = 440), followed by women from Western Africa (n = 326), Southern Africa (n = 207) and Northern Africa (n = 46). Of the women tested for an HPV infection in Middle Africa, none were diagnosed with ASCUS (**Table 1**).

HPV16, 52, 18, 35, 45, 51, 66, 53, 39 and 58 were the ten most common genotypes in women with ASCUS (**Table 2**). The prevalence of type-specific HPV and distribution in women diagnosed with ASCUS for each geographic region is presented in **Fig 2**, in the second panel from the left.

For LSIL, data from a total of 1,444 women from 23 studies were abstracted. A majority of the patients were from Eastern (n = 605), followed by Southern (n = 521), Western (n = 286, Northern (n = 24) and Middle (n = 8) (**Table 1**) Africa regions. HPV16, 35, 52, 18, 53, 56, 58, 51, 45 and 66 were the top ten most common genotypes in women with LSIL (**Table 2**). HPV type specific prevalence and distribution by region for LSIL is summarized in (**Fig 2**, in the third panel from the left).

### Prevalence of HPV genotypes in high-grade intraepithelial lesions

Guan et al. [21] attempted to analyze African HPV genotype prevalence in high-grade lesions, where only 185 out of 245 patient samples tested positive for HPV. In our systematic review, 33 studies comprising a total of 1,571 HSIL cases were included in the analysis. A majority of HSIL cases came from Southern (n = 768), followed by Eastern (n = 563), Western (n = 102), and Middle (n = 19) regions of Africa. Only 8 and 17 cases were from the Northern region and mixed region, respectively.

HPV16, 18, 35, 52, 58, 33, 31, 53, 45 and 66 were the ten most frequently detected genotypes in HSIL cases (**Table 2**). HPV type-specific prevalence and distribution by region for HSIL is summarized in (**Fig 2**). Western Africa reported the highest prevalence of HPV16 and 18 at 33% and 22.2% respectively for HSIL. In Eastern and Southern regions, HPV16 prevalence was tied at 28%, followed by HPV 52 (16.5%) and HPV 35 (15.1%), respectively.

Overall, 30 studies stratified eligible women screened for HPV infection by age. Analysis of these studies revealed that the age group 45–54 years had the highest overall prevalence of any HPV for HSIL at 87.1% (95% CI: (67.3–107.0; I^2^ = 0.0%) as shown in **Table 3**.

### Prevalence of HPV genotypes in cervical cancer

A total of 4,067 cases from 43 studies were included in the ICC systematic review analysis (**Table 1**). A majority of the ICC cases were from the Eastern region (n = 1,905), followed by Western (n = 951), Northern (n = 517), Southern (n = 457) regions, and only three patients were from the Middle Africa region. Two hundred and thirty-five patients were from mixed regions.

HPV16, 18, 45, 35, 33, 52, 31, 58, 51 and 68 were the top ten most frequently detected genotypes among ICC cases (**Table 2**). While HPV16, 18, 35, and 52 were the four most common types in normal cytology through HSIL lesions; in ICC tissues HPV45 replaced HPV52 as the third most common genotype (**Table 2**). Together HPV16, 18, and 45 accounted for 77.7% of all ICC cases in African women.

HPV type-specific prevalence and distribution by region is presented in **Fig 2**. The prevalence of HPV16, 18, and 45 in ICC cases were 49.7% (95% CI: 48.2–51.2; I^2^ = 88.6%), 18.0% (95% CI: 16.8–19.2; I^2^ = 78.2%) and 10.0% (95% CI: 9.1–10.9; I^2^ = 85.7%), respectively. HPV16 and 18 varied by region; in descending order: Northern (61%, and 22.4%), Eastern (54.4% and 21.0%), Southern (46.8% and 18%), and Western (44.1% and 11.7%) regions of Africa.

## Discussion

Comprehensive data on the geographic distribution of specific HPV genotypes in women with all grades of cervical diagnosis are crucial for estimating the baseline burden of disease, the future impact of HPV vaccines on cervical cancer prevention, and the identification of optimal HPV screening tools. To the best of our knowledge, our report is the largest meta-analysis to date of HPV genotype distribution and prevalence in women from Africa by region, lesion type, age group, publication year, and PCR primers for genotyping. Seventy-one studies matched the inclusion criteria and were abstracted for systematic review and meta-analysis. Each of the five sub-regions of African countries (n = 23) was fairly represented with the exception of the Middle region, where only two studies were included.

Overall, the prevalence of any HPV infection in Africa was higher than other world regions; our meta-analysis determined the prevalence rates in women with normal cervical cytology (29.3%), ASCUS (46.5%), LSIL (74.2%), HSIL (84.8%) and ICC (89.5%) [21]. Our study revealed that by pooling several studies examining HPV infection among women with normal cervical cytology, Southern Africa has the highest of any HPV infections at 57.3%, followed by Eastern Africa (42.2%), Western Africa (27.8%) and Northern Africa (12.8%). The prevalence of any HPV infection increased among women by cervical disease grade in all regions, except Southern Africa. However, the inclusion in this analysis of various genotyping techniques with varying sensitivities might impact the accuracy of HPV specific genotype prevalence.

Nevertheless, the four most common genotypes, HPV16, 18, 52 and 35, were identical from normal cervical cytology through HSIL. In ICC cases, however, HPV45 replaced HPV52 as the third most common genotype. The high prevalence rate of HPV45 in Africa was previously reported among ICC cases [18,19,22,110,111]. Further analysis of women diagnosed with ICC by region confirmed that despite the differences in prevalence of HPV16, 18 and 45, these three genotypes remained the most common in all regions for ICC. HPV33 was the fourth most common in all regions except in Western Africa, where it is HPV35. These findings suggest a higher prevalence of HPV45 in ICC and HPV52 in HSIL than previously found [23]. The prevalence of HPV16 and 18 were much greater in ICC cases than in HSIL cases (49.7% vs. 31.2% and 18.0% vs. 13.9%, respectively). The combined prevalence of HPV16 and 18 in cases of ICC is 67.7%, in agreement with the ~70% global estimate found by other meta-analyses [19,21–23].

While there has been a substantial push toward mass vaccination with either Gardasil or Cervarix [112], until now there was insufficient data to demonstrate the high prevalence of HPV16/18 in cases of ICC in all sub-regions of the African continent. Data from the present study indicate that vaccines currently available could prevent nearly 70% of cervical cancer cases in all sub-regions of Africa. Moreover, a polyvalent vaccine in Phase III clinical trials known to protect against HPV16, 18, 31, 33, 35, 45, 51, 52, and 58 could prevent nearly all cases of cervical cancer in African women. Unfortunately, because most African countries are currently unprepared to implement HPV vaccination, even the availability of this nonavalent vaccine would leave an entire generation unprotected in Africa [113]. These barriers include the long duration of time it would take for the vaccine to be ready for mass distribution, the negotiation of its cost, and the placement of infrastructure to deliver the vaccine to adolescent girls at risk of developing cervical cancer [13,114]. Thus, other cancer prevention tools such as cervical screening through visual inspection with acetic acid and/or Lugol’s iodine, or detection of high-risk HPV genotypes may help reduce the high burden of cervical cancer morbidity and mortality in the region.

Using the stringent inclusion and exclusion criteria outlined in our meta-analysis, six studies from Northern Africa qualified for data abstraction for women with normal cervical cytology (n = 1,865) and ICC (n = 517). Notably, 83.4% of ICC cases were associated with either HPV16 (61%) or 18 (22.4%) in the sub-region, the highest number ever reported from any part of the globe. However, the prevalence of any HPV infection among women with normal cytology was low (12.8%) compared to other regions which ranged between 27.8–57.3%. The prevalence of HPV16 and 18 is 2.9% and 0.9%, respectively. Since most of the women screened for cervical cancers are adults over 18 years of age, low prevalence of HPV16 and 18 in the population suggests that there is a wider window available for delivering HPV vaccine, particularly if it is combined with HPV DNA screening among older women. A recent review on the burden of HPV infections and related diseases in the extended Middle East and Northern Africa reported very low prevalence of HPV infection in women with normal cervical cytology, similar to current findings [115]. However, our study revealed a higher prevalence of HPV16/18 among women diagnosed with ICC than previously reported [115]. The high prevalence of HPV16/18 in ICC in Northern Africa requires urgent attention such as encouraging the use of culturally acceptable tools to increase cervical cancer screenings and HPV DNA testing among the population so as to have a complete picture of the prevalence of HPV genotypes by all stages of cervical cancer grade. Due to limited data, our study was unable to yield conclusive HPV prevalence in women from Northern Africa diagnosed with ASCUS, LSIL and HSIL cervical lesions, due to limited data. Of great concern to policy makers in Northern Africa region is that very few interventions to create awareness about cervical cancer, implementation of cervical cancer screening programs, HPV DNA testing, or use of prophylactic vaccines to prevent HPV infection have been reported.

A majority of the population in Northern Africa share similar cultural and religious traditions and known to have more conservative views towards sexual behavior than countries in sub-Saharan Africa. If indeed future studies in Northern Africa clearly indicate that a majority of women in the region with normal cervical cytology have lower risk of any HPV infection even at advanced ages (18–26 years), then it will be important to increase the age of vaccination for Northern African girls up from the current WHO recommended 9–13 years to a much older age [116]. The legal adult age is between 16–18 years in most countries, which could serve as a more appropriate vaccination age for the region.

The increasing availability of additional tools to prevent and fight cervical cancer provides an opportunity for all nations to implement cervical cancer control programs that are both cost-effective and easier to deliver or to improve existing programs by saving additional lives and reducing cost [117]. The GAVI Alliance, in an effort to close the gap in access to life-saving vaccines [28], recently announced a deal with pharmaceutical industries to offer the HPV vaccine at US $4.50 per dose [28,29] to developing countries meeting GAVI’s eligibility criteria [13]. To date, 19 sub-Saharan African countries have been approved to pilot HPV vaccine demonstration projects, however, despite the high prevalence of HPV16/18 in ICC cases in Northern Africa, not a single country from the region has been approved for HPV vaccine support [29].

The implementation of one lifetime screening at age 35 with either visual inspection with acetic acid or HPV DNA testing would reduce the risk of developing cervical cancer by 25% and cost less than $500 per person per year of life saved [11]. However, the lack of laboratory infrastructure for HPV genotyping in nearly the entire African continent, with the exception of South Africa, Morocco and Ethiopia as shown in our study, should be a major concern to all who are working to fight cervical cancer in Africa. In our systematic review, the majority of the HPV DNA genotyping (for the studies eligible for data abstraction) was performed outside of Africa in the USA, Japan or Europe. HPV DNA testing is currently the most sensitive and reproducible cervical screening test [118], therefore, there is an urgent need to build the infrastructure in Africa to facilitate efficient cervical screening.

The major contribution of our study to the current body of literature is the assessment of 25,463 women from all African regions of whom 46.8% were HPV infected. A further stratification of specific HPV genotype prevalence by disease severity will help identify appropriate tools for HPV genotyping and assess the future impact of HPV vaccines. However, the applicability of this data throughout Africa is limited, since 95% of the studies were obtained from Eastern, Western and Southern African regions, partially attributable to the lack of well-designed studies in the remainder of Africa, especially Middle and Northern Africa. Indeed, quality studies meeting stringent meta-analysis criteria for inclusion were missing from 30 African countries, thus future cervical cancer prevention policies should consider building laboratory infrastructure in these countries.

The current WHO recommendations on cervical cancer screening are based on age [119], however, only a few studies stratified the population studied by age. A majority of studies reported mean or median age, which is not helpful in defining the prevalence of HPV infection by age-group to determine an appropriate age to target for HPV DNA testing or cervical cancer screening. This consideration should be factored in the design of future studies in the continent.

Some of the limitations to our study include: (i) the inclusion of cross-sectional studies and their inherent risk of bias; (ii) inclusion of studies that over sampled HIV-seropositive patients in the analysis; and (iii) there may be lack of representativeness of HPV type specific prevalence due to variation in different genotyping methods used and tissue sources. Finally, while we made every effort to identify and include in our meta-analysis all eligible studies, it is possible that we missed some.

Although previous research has established the relationship between HIV and HPV infection, the inter-relationship is not fully understood [63,102,120]. Of the estimated 35.3 million individuals worldwide living with HIV/AIDS in 2012, approximately 70% are living in Africa [121]. Women make up 60% of the HIV-infected population in Africa [121], and nearly 40% of HIV seropositive individuals without cervical abnormalities test positive for HPV infection [110]. Studies are underway in our group to specifically estimate the prevalence of HPV genotypes among women living with HIV in Africa.

## Supporting Information

S1 Appendix A detailed search strategy. (DOC)

S2 Appendix PROSPERO International prospective register of systematic reviews registration. (PDF)

S1 Table PRISMA checklist for reporting systematic reviews and meta-analysis (DOC)

S2 Table Methodological quality assessment and list of articles included in the study. (DOCX)

S3 Table Study characteristics and HPV prevalence by country and study. (DOCX)

S4 Table Prevalence of any HPV by tissue source, country and GNI. (PDF)
