# Supplementary material for: Prevalence of Human Papillomavirus Genotypes among African Women with Normal Cervical Cytology and Neoplasia: A Systematic Review and Meta-Analysis
Source: PLoS One. 2015 Apr 14;10(4):e0122488. doi: 10.1371/journal.pone.0122488 (PMC4396854; doi:10.1371/journal.pone.0122488)
Supplement: S1 Appendix — (DOC) [file pone.0122488.s001.doc]

**Appendix S1: Search strategies for electronic databases**

**PubMed/MEDLINE (NCBI)**481 records returned on Aug. 27, 2013

(Papillomaviridae[Mesh] OR Papillomavirus Vaccines[Mesh] OR alphapapilloma*[tiab] OR betapapilloma*[tiab] OR gammapapilloma*[tiab] OR mupapilloma*[tiab] OR papilloma*[tiab] OR HPV*[tiab])

AND

(Cervical[tiab] OR cervix[tiab] OR uter*[tiab])

AND

(Uterine Cervical Dysplasia[Mesh] OR Uterine Cervical Neoplasms[Mesh] OR HSIL*[tiab] OR high grade squamous intraepithelial[tiab] OR cancer*[tiab] OR tumor[tiab] OR tumors[tiab] OR tumoral*[tiab] OR neoplas*[tiab] OR tumour*[tiab] OR dysplasia*[tiab] OR dysplastic[tiab] OR carcino*[tiab] OR adenosquam*[tiab] OR adenocarcinoma*[tiab])

AND

("Africa South of the Sahara"[Mesh] OR africa[all fields] OR southern africa[all fields] OR west africa[all fields] OR western africa[all fields] OR central africa[all fields] OR west african[all fields] OR east african[all fields] OR south african[tiab] OR central african[tiab] OR Cameroon[all fields] OR Central African Republic[all fields] OR Chad[all fields] OR Congo[all fields] OR Democratic Republic of the Congo[all fields] OR Equatorial Guinea[all fields] OR Gabon[all fields] OR Burundi[all fields] OR Djibouti[all fields] OR Eritrea[all fields] OR Ethiopia[all fields] OR Kenya[all fields] OR Rwanda[all fields] OR Somalia[all fields] OR Sudan[all fields] OR Tanzania[all fields] OR Uganda[all fields] OR Angola[all fields] OR Botswana[all fields] OR Lesotho[all fields] OR Malawi[all fields] OR Mozambique[all fields] OR Namibia[all fields] OR South Africa[all fields] OR Swaziland[all fields] OR Zambia[all fields] OR Zimbabwe[all fields] OR Benin[all fields] OR Burkina Faso[all fields] OR Cape Verde[all fields] OR Cote d'Ivoire[all fields] OR "ivory coast"[all fields] OR Gambia[all fields] OR Ghana[all fields] OR Guinea[all fields] OR Guinea-Bissau[all fields] OR Liberia[all fields] OR Mali[all fields] OR Mauritania[all fields] OR Niger[all fields] OR Nigeria[all fields] OR Senegal[all fields] OR Sierra Leone[all fields] OR Togo[all fields])

**Embase (Elsevier):**705 records returned on Aug. 27, 2013

('papilloma virus'/exp OR 'wart virus vaccine'/exp OR alphapapilloma*:ab,ti OR betapapilloma*:ab,ti OR gammapapilloma*:ab,ti OR mupapilloma*:ab,ti OR papilloma*:ab,ti OR hpv*:ab,ti)

AND

(cervical:ab,ti OR cervix:ab,ti OR uter*:ab,ti)

AND

('uterine cervix dysplasia'/exp OR 'uterine cervix tumor'/exp OR HSIL*:ti,ab OR high grade squamous intraepithelial:ti,ab OR cancer*:ti,ab OR tumor:ti,ab OR tumors:ti,ab OR tumoral*:ti,ab OR tumour*:ti,ab OR dysplasia*:ti,ab OR dysplastic:ti,ab OR neoplas*:ti,ab OR carcino*:ti,ab OR adenosquam*:ti,ab OR adenocarcinoma*:ti,ab)

AND

('Africa south of the Sahara'/exp OR 'africa' OR 'southern africa' OR 'west africa' OR 'western africa' OR 'central africa' OR 'east african' OR 'west african' OR 'south african' OR 'central african' OR 'Cameroon' OR 'Central African Republic' OR 'Chad' OR 'Congo' OR 'Democratic Republic of the Congo' OR 'Equatorial Guinea' OR 'Gabon' OR 'Burundi' OR 'Djibouti' OR 'Eritrea' OR 'Ethiopia' OR 'Kenya' OR 'Rwanda' OR 'Somalia' OR 'Sudan' OR 'Tanzania' OR 'Uganda' OR 'Angola' OR 'Botswana' OR 'Lesotho' OR 'Malawi' OR 'Mozambique' OR 'Namibia' OR 'South Africa' OR 'Swaziland' OR 'Zambia' OR 'Zimbabwe' OR 'Benin' OR 'Burkina Faso' OR 'Cape Verde' OR 'Cote d Ivoire' OR 'Cote dIvoire' OR 'ivory coast' OR 'Gambia' OR 'Ghana' OR 'Guinea' OR 'Guinea-Bissau' OR 'Liberia' OR 'Mali' OR 'Mauritania' OR 'Niger' OR 'Nigeria' OR 'Senegal' OR 'Sierra Leone' OR 'Togo')

**Web of Science, including Science Citation Index, Social Science Citation Index, and Arts and Humanities Citation Index (Thomson Reuters)**468 records returned on Aug. 27, 2013

**BIOSIS Previews (Thomson Reuters)**267 records returned on Aug. 27, 2013

The same search was run on the Web of Knowledge platform for both databases.

TS=(alphapapilloma* OR betapapilloma* OR gammapapilloma* OR mupapilloma* OR papilloma* OR HPV*)

AND

TS=(cervical OR cervix OR uter*)

AND

TS=(HSIL* OR "high grade squamous intraepithelial" OR cancer* OR tumor OR tumors OR tumoral* OR tumour* OR dysplasia* OR dysplastic OR neoplas* OR carcino* OR adenosquam* OR adenocarcinoma*)

AND

TS=(africa OR "southern africa" OR "west africa" OR "western africa" OR "west african" OR "east african" OR "south african" OR "central africa" OR "central african" OR Cameroon OR "Central African Republic" OR Chad OR Congo OR "Equatorial Guinea" OR Gabon OR Burundi OR Djibouti OR Eritrea OR Ethiopia OR Kenya OR Rwanda OR Somalia OR Sudan OR Tanzania OR Uganda OR Angola OR Botswana OR Lesotho OR Malawi OR Mozambique OR Namibia OR "South Africa" OR Swaziland OR Zambia OR Zimbabwe OR Benin OR "Burkina Faso" OR "Cape Verde" OR "Cote d'Ivoire" OR "ivory coast" OR Gambia OR Ghana OR Guinea OR "Guinea-Bissau" OR Liberia OR Mali OR Mauritania OR Niger OR Nigeria OR Senegal OR "Sierra Leone" OR Togo)

**POPLINE (K4Health)**159 records returned on Aug. 27, 2013

(alphapapillomavirus OR betapapillomavirus OR gammapapillomavirus OR mupapillomavirus OR papillomavirus OR papilloma OR alphapapillomaviruses OR betapapillomaviruses OR gammapapillomaviruses OR mupapillomaviruses OR papillomaviruses OR HPV)

AND

(africa OR "southern africa" OR "west africa" OR "western africa" OR "central africa" OR "west african" OR "east african" OR "south african" OR "central african" OR Cameroon OR "Central African Republic" OR Chad OR Congo OR "Democratic Republic of the Congo" OR "Equatorial Guinea" OR Gabon OR Burundi OR Djibouti OR Eritrea OR Ethiopia OR Kenya OR Rwanda OR Somalia OR Sudan OR Tanzania OR Uganda OR Angola OR Botswana OR Lesotho OR Malawi OR Mozambique OR Namibia OR South Africa OR Swaziland OR Zambia OR Zimbabwe OR Benin OR "Burkina Faso" OR "Cape Verde" OR "Cote d'Ivoire" OR "ivory coast" OR Gambia OR Ghana OR Guinea OR "Guinea-Bissau" OR Liberia OR Mali OR Mauritania OR Niger OR Nigeria OR Senegal OR Sierra Leone OR Togo) AND (Cervical OR cervix OR uter*) AND (HSIL* OR cancer* OR tumor OR tumors OR tumour OR tumours OR tumoral* OR neoplas* OR carcino* OR adenosquam* OR adenocarcinoma*)

**Cochrane Central Register of Controlled Trials (Wiley)**13 records returned on Aug. 27, 2013

(alphapapilloma* OR betapapilloma* OR gammapapilloma* OR mupapilloma* OR papilloma* OR HPV*)

AND

Cervical OR cervix OR uter*

AND

(HSIL* OR cancer* OR tumor OR tumors OR tumour OR tumours OR tumoral* OR neoplas* OR carcino* OR adenosquam* OR adenocarcinoma*)

AND

(africa OR "southern africa" OR "west africa" OR "western africa" OR "central africa" OR "west african" OR "east african" OR "south african" OR "central african" OR Cameroon OR "Central African Republic" OR Chad OR Congo OR "Democratic Republic of the Congo" OR "Equatorial Guinea" OR Gabon OR Burundi OR Djibouti OR Eritrea OR Ethiopia OR Kenya OR Rwanda OR Somalia OR Sudan OR Tanzania OR Uganda OR Angola OR Botswana OR Lesotho OR Malawi OR Mozambique OR Namibia OR South Africa OR Swaziland OR Zambia OR Zimbabwe OR Benin OR "Burkina Faso" OR "Cape Verde" OR "Cote d'Ivoire" OR "ivory coast" OR Gambia OR Ghana OR Guinea OR "Guinea-Bissau" OR Liberia OR Mali OR Mauritania OR Niger OR Nigeria OR Senegal OR Sierra Leone OR Togo)

**Dissertations and Theses Full Text (ProQuest)**22 records returned on Aug. 27, 2013

ALL(alphapapilloma* OR betapapilloma* OR gammapapilloma* OR mupapilloma* OR papilloma* OR HPV*)

AND

ALL(Cervical OR cervix OR uter*)

AND

ALL(HSIL* OR cancer* OR tumor OR tumors OR tumour OR tumours OR tumoral* OR neoplas* OR carcino* OR adenosquam* OR adenocarcinoma*)

AND

ALL(africa OR "southern africa" OR "west africa" OR "western africa" OR "central africa" OR "west african" OR "east african" OR "south african" OR "central african" OR Cameroon OR "Central African Republic" OR Chad OR Congo OR "Democratic Republic of the Congo" OR "Equatorial Guinea" OR Gabon OR Burundi OR Djibouti OR Eritrea OR Ethiopia OR Kenya OR Rwanda OR Somalia OR Sudan OR Tanzania OR Uganda OR Angola OR Botswana OR Lesotho OR Malawi OR Mozambique OR Namibia OR South Africa OR Swaziland OR Zambia OR Zimbabwe OR Benin OR "Burkina Faso" OR "Cape Verde" OR "Cote d'Ivoire" OR "ivory coast" OR Gambia OR Ghana OR Guinea OR "Guinea-Bissau" OR Liberia OR Mali OR Mauritania OR Niger OR Nigeria OR Senegal OR Sierra Leone OR Togo)\

**African Index Medicus (WHO)**13 records returned on Aug. 27, 2013

alphapapillomavirus OR betapapillomavirus OR gammapapillomavirus OR mupapillomavirus OR papillomavirus OR papilloma OR alphapapillomaviruses OR betapapillomaviruses OR gammapapillomaviruses OR mupapillomaviruses OR papillomaviruses OR HPV
