# Supplementary material for: Prevalence of Human Papillomavirus Genotypes among African Women with Normal Cervical Cytology and Neoplasia: A Systematic Review and Meta-Analysis
Source: PLoS One. 2015 Apr 14;10(4):e0122488. doi: 10.1371/journal.pone.0122488 (PMC4396854; doi:10.1371/journal.pone.0122488)
Supplement: S2 Appendix — (PDF) [file pone.0122488.s002.pdf]

UNIVERSITY of York
Centre for Reviews and Dissemination

NHS
National Institute for Health Research

PROSPERO International prospective register of systematic reviews

Human papillomavirus genotype prevalence among African women with normal cervical cytologies and lesions: a systematic review and meta-analysis

Javier Ogembo, Paul Bain, Alaina Seymour, Henry Park

Citation

Javier Ogembo, Paul Bain, Alaina Seymour, Henry Park. Human papillomavirus genotype prevalence among African women with normal cervical cytologies and lesions: a systematic review and meta-analysis. PROSPERO 2013:CRD42013006558 Available from http://www.crd.york.ac.uk/PROSPERO/display_record.asp?ID=CRD42013006558

Review question(s)

What HPV genotypes are predominant in African women with normal and different cervical lesions?
What HPV genotypes are predominant in HIV positive African women with normal and different cervical lesions?

Searches

PubMed/MEDLINE (NCBI), Embase (Elsevier), Web of Science (Thomson), BIOSIS Preview (Thomson), Dissertations and Theses (ProQuest), Cochrane Central Register of Controlled Trials (Wiley), African Index Medicus (WHO), and POPLINE (K4Health). We also attempted to identify unpublished studies by examining ClinicalTrials.gov (NIH), International Clinical Trial Registry Platform (WHO), European Union Clinical Trials Register (EMA), and System for Information on Grey Literature in Europe (OpenGrey).

Types of study to be included

Observational studies or control arms of randomized controlled trials with individual level data will be assessed by a checklist of essential items as outlined in STROBE by three reviewers.

Condition or domain being studied

Human papillomavirus, the causal organism of cervical cancer in adult women.

Participants/ population

All African adult women screened for different HPV genotypes.

Intervention(s), exposure(s)

A comprehensive strategy was used to identify studies where the physical document and relevant data were accessible, studies including women living in African countries. The study should have included at least 10 women from sub-Saharan Africa, cervical cytology/histology was confirmed by fixed/fresh biopsy or exfoliated cervical cells, the study included HPV genotype-specific prevalence on at least types 16, 18, and one other genotype, and genotype prevalence was stratified by cervical histology/grade.

Comparator(s)/ control

Comparisons will be made within the groups e.g. African regions as per WHO classification, country, type of lesions, tissue biopsies, methods of genotyping.

Context

Studies must have included adult women living in the African continent.

Outcome(s)

Primary outcomes

Determine the prevalence of HPV genotypes in African women with normal and cervical cytologies. This will help inform whether the existing two prophylactic vaccines currently used in developed countries will have the same impact in the continent.
Open.

Secondary outcomes

Will help inform whether the existing two prophylactic vaccines currently used in developed countries will have the same impact in the continent.
Open.

Data extraction, (selection and coding)
Three separate reviewers (R.K.O, A.J.S. and J.G.O.) read the titles and abstracts to determine each study's eligibility using the following pre-specified criteria for inclusion: the physical document and relevant data were accessible. Discrepancies were resolved by consensus.

Risk of bias (quality) assessment
Funnel plots will be used to assess the risk of bias of cross-sectional studies.

Strategy for data synthesis
Data will be grouped based on country where the study was conducted, geographical region as outlined by WHO, type of biopsies analyzed, methods of screening HPV genotypes.

Analysis of subgroups or subsets
High risk vs Low risk HPV genotypes

Dissemination plans
Publication in peer-reviewed journal

Contact details for further information
Javier Ogembo
364 Plantation Street, LRB Room 211
Worcester, MA 01605
Phone: 508-856-3380
javier.ogembo@umassmed.edu

Organisational affiliation of the review
University of Massachusetts Medical School
www.umassmed.edu

Review team
Dr Javier Ogembo, University of Massachusetts Medical School
Paul Bain, Countway Library of Harvard Medical School
Alaina Seymour, Northeastern University
Henry Park, Yale University

Anticipated or actual start date
01 October 2013

Anticipated completion date
30 January 2014

Funding sources/sponsors
None

Conflicts of interest
None known

Language
English

Country
United States of America

Subject index terms status
Subject indexing assigned by CRD

Subject index terms
Africa; Alphapapillomavirus; Genotype; Humans; Papillomavirus Infections; Prevalence;

Stage of review
Ongoing

Date of registration in PROSPERO
29 November 2013

Date of publication of this revision
29 November 2013

**Stage of review at time of this submission**	**Started**	**Completed**

Preliminary searches	Yes	No
Piloting of the study selection process	Yes	No
Formal screening of search results against eligibility criteria	No	No
Data extraction	No	No
Risk of bias (quality) assessment	No	No
Data analysis	No	No

PROSPERO
This information has been provided by the named contact for this review. CRD has accepted this information in good faith and registered the review in PROSPERO. CRD bears no responsibility or liability for the content of this registration record, any associated files or external websites.
