# Supplementary material for: Prevalence of Human Papillomavirus Genotypes among African Women with Normal Cervical Cytology and Neoplasia: A Systematic Review and Meta-Analysis
Source: PLoS One. 2015 Apr 14;10(4):e0122488. doi: 10.1371/journal.pone.0122488 (PMC4396854; doi:10.1371/journal.pone.0122488)
Supplement: S2 Table — (DOCX) [file pone.0122488.s004.docx]

**Table S2** – Quality Assessment Tools Answers

| **Study** | | Q1: Did the study attempt to address non-response bias? | | Q2: Was the study free from outcome reporting bias? | Q3: Did the study report response rate? | Q4: Did the study use a representative sampling strategy? | Q5: Did the study use adequate time of sampling? | Q6: Did the study use sensitive sampling techniques? | Q7: Was the study free from conflict(s) of interest? |
| --- | --- | --- | --- | --- | --- | --- | --- | --- | --- |
| Abate et al. 2013a [1] | | NA | | Yes | NA | Yes | No | Yes | Yes |
| Adjorlolo-Johnson et al. 2010 [2] | | Unclear | | Yes | Yes | Yes | Yes | Yes | Yes |
| Alhamany et al. 2010 [3] | | Unclear | | Unclear | Unclear | Unclear | Unclear | Yes | Yes |
| Allan et al. 2008 [4] | | Unclear | | Yes | Unclear | Unclear | Unclear | Yes | No |
| Astori et al. 1999 [5] | | Unclear | | Yes | Yes | No | Yes | Yes | Yes |
| Bayo et al. 2002 [6] | | Unclear | | Yes | Yes | Yes | Yes | Yes | Unclear |
| Bekele et al. 2010 [7] | | NA | | Yes | NA | Unclear | No | Yes | Yes |
| Blossom et al. 2007 [8] | | Unclear | | Yes | Yes | Unclear | Unclear | Yes | Yes |
| Bosch et al. 1995 [9] | | Unclear | | Yes | No | Unclear | Yes | Yes | Unclear |
| Castellsague et al. 2008 [10] | | Unclear | | Yes | Yes | Yes | Yes | Yes | Unclear |
| Chabaud et al. 1996 [11] | | Unclear | | Yes | Unclear | No | Yes | Yes | Yes |
| Chaouki et al. 1998 [12] | | Unclear | | Yes | Yes | Yes | Yes | Yes | Yes |
| Cooper et al. 1991a [13] | | NA | | Yes | NA | Unclear | No | No | Yes |
| Cooper et al. 1991b [14] | | NA | | Yes | NA | Unclear | No | No | Yes |
| Czegledy et al. 1992 [15] | | Unclear | | Yes | Unclear | Unclear | No | Yes | Yes |
| Dartell et al. 2012 [16] | | Unclear | | Yes | Yes | Yes | Yes | Yes | No |
| De Vuyst et al. 2003 [17] | | Unclear | | Yes | Yes | Unclear | Yes | Yes | No |
| De Vuyst et al. 2008 [18] | | Unclear | | Yes | Yes | Unclear | Yes | Yes | No |
| De Vuyst et al. 2010 [19] | | Unclear | | Yes | Yes | Unclear | Yes | Yes | Yes |
| De Vuyst et al. 2011 [20] | | Unclear | | Yes | Yes | No | Yes | Yes | Yes |
| De Vuyst et al. 2012 [21] | | Unclear | | Yes | Yes | No | Yes | Yes | Yes |
| Denny et al. 2008 [22] | | Unclear | | Yes | Yes | No | Yes | Yes | No |
| Denny et al. 2014 [23] | | Unclear | | Yes | Yes | No | Yes | Yes | No |
| Dols et al. 2012 [24] | | Unclear | | No | Unclear | No | Yes | Yes | Yes |
| Fanta, 2005 [25] | | NA | | Unclear | NA | Unclear | No | Yes | Yes |
| Firnhaber et al. 2009 [26] | | Unclear | | No | Yes | No | Yes | Yes | No |
| Firnhaber et al. 2010 [27] | | Unclear | | No | Yes | No | Yes | Yes | Yes |
| Gage et al. 2012 [28] | | Yes | | Yes | Yes | Yes | Yes | Yes | Yes |
| Garcia-Espinosa et al. 2009 [29] | | Unclear | | Yes | Yes | Yes | Yes | Yes | Yes |
| Gravitt et al. 2002 [30] | | Unclear | | Yes | Yes | Yes | Yes | Yes | Yes |
| Hammouda et al. 2005 [31] | | Unclear | | Yes | Unclear | Unclear | Yes | Yes | Unclear |
| Hammouda et al. 2011 [32] | | Yes | | Unclear | Yes | Yes | Yes | Yes | Yes |
| Icenogle et al. 1992 [33] | | Unclear | | Unclear | Unclear | No | Yes | Yes | Yes |
| Jaquet et al. 2012 [34] | | Unclear | | Unclear | Yes | No | Yes | Yes | Yes |
| Jones et al. 2007 [35] | | Unclear | Yes | Yes | Unclear | Yes | Yes | Yes |  |
| Kay et al. 2003 [36] | | NA | Yes | NA | No | Yes | Yes | Yes |  |
| Keita et al. 2009 [37] | | Yes | Yes | Yes | Yes | Yes | Yes | Yes |  |
| La Ruche et al. 1998 [38] | | Unclear | Yes | Yes | Unclear | Yes | Yes | Yes |  |
| MacLeod et al. 2011 [39] | | Unclear | Yes | Yes | No | Yes | Yes | Yes |  |
| Marais et al. 2000 [40] | | Unclear | Yes | Yes | No | Yes | Yes | Unclear |  |
| Marais et al. 2008 [41] | | Unclear | Yes | Yes | Yes | Yes | Yes | Unclear |  |
| Maranga et al. 2013 [42] | | Unclear | Yes | Yes | Unclear | Yes | Yes | Yes |  |
| Mayaud et al. 2003 [43] | | Unclear | Yes | Yes | Unclear | Yes | Yes | Yes |  |
| McDonald et al. 2012 [44] | | Yes | No | Yes | Yes | Yes | Yes | No |  |
| Mzibiri et al. 2009 [45] | | Unclear | Unclear | Unclear | Unclear | Unclear | Yes | Yes |  |
| Moodley et al. 2009 [46] | | Unclear | Yes | Yes | Unclear | Yes | Yes | Yes |  |
| Naucler et al. 2004 [47] | | Unclear | Yes | Yes | No | Unclear | Yes | Yes |  |
| Naucler et al. 2011 [48] | | Unclear | Yes | Yes | No | Yes | Yes | Yes |  |
| Ndiaye et al. 2012 [49] | | NA | Yes | NA | Unclear | Unclear | Yes | Yes |  |
| Ng'andwe et al. 2007 [50] | | Unclear | Yes | Unclear | Unclear | Yes | Yes | Yes |  |
| Odida et al., 2008 [51] | | NA | Yes | NA | No | No | Unclear | Yes |  |
| Odida et al. 2010 [52] | | NA | Yes | NA | Unclear | No | Yes | Yes |  |
| Odida et al. 2011 [53] | | Unclear | Yes | Yes | Yes | Yes | Yes | Yes |  |
| Okolo et al. 2010 [54] | | NA | Yes | NA | Unclear | No | Yes | Yes |  |
| Piras et al. 2011 [55] | | Yes | Yes | Yes | Yes | Yes | Yes | Yes |  |
| Rahman et al. 2011 [56] | | No | Yes | Unclear | No | Yes | Yes | Yes |  |
| Ramesar et al. 1996 [57] | | Unclear | Yes | Yes | No | Yes | No | Yes |  |
| Ramogola-Masire et al. 2011[58] | | Unclear | Yes | Unclear | No | Yes | Yes | Yes |  |
| Richter et al. 2008 [59] | | Unclear | Yes | Yes | No | Yes | Yes | Yes |  |
| Rogo, 1990 [60] | | na | Yes | na | No | No | Yes | Yes |  |
| Sahasrabuddhe et al. 2007 [61] | | Unclear | Yes | Yes | No | Yes | Yes | Yes |  |
| Said et al. 2009 [62] | | Unclear | Yes | Unclear | Yes | Yes | Yes | Yes |  |
| Singh et al. 2009 [63] | | Unclear | Yes | Yes | No | Yes | Yes | Yes |  |
| Stanczuk et al. 2003a [64] | | Unclear | Yes | Yes | No | Yes | Yes | Yes |  |
| Stanczuk et al. 2003b [65] | | Unclear | Yes | Yes | No | Yes | Yes | Yes |  |
| Thomas et al. 2004 [66] | | Unclear | Yes | Yes | Yes | Yes | Yes | Yes |  |
| Vidal et al. 2011 [67] | | Unclear | Yes | Yes | Yes | Yes | Yes | Yes |  |
| Wall et al. 2005 [68] | |  |  |  |  |  |  |  |  |
| Williamson et al. 1989 [69] | | Unclear | Yes | Yes | No | Yes | No | Yes |  |
| Williamson et al. 1994 [70] | | NA | Yes | NA | No | NA | No | Yes |  |
| Xi et al. 2003 [71] | | Unclear | Yes | Yes | Yes | Yes | Yes | Yes |  |

**References**

1. Abate E, Aseffa A, El-Tayeb M, El-Hassan I, Yamuah L, et al. (2013) Genotyping of human papillomavirus in paraffin embedded cervical tissue samples from women in Ethiopia and the Sudan. J Med Virol 85: 282-287.

2. Adjorlolo-Johnson G, Unger E, Boni-Ouattara E, Touré-Coulibaly K, Maurice C, et al. (2010) Assessing the relationship between HIV infection and cervical cancer in Côte d'Ivoire A case-control study. BMC infectious diseases 10: 242.

3. Alhamany Z, El Mzibri M, Kharbach A, Malihy A, Abouqal R, et al. (2010) Prevalence of human papillomavirus genotype among Moroccan women during a local screening program. The Journal of Infection in Developing Countries 4: 732-739.

4. Allan B, Marais DJ, Hoffman M, Shapiro S, Williamson AL (2008) Cervical human papillomavirus (HPV) infection in South African women: implications for HPV screening and vaccine strategies. Journal of clinical microbiology 46: 740-742.

5. Astori G, Beltrame A, Pipan C, Raphenon G, Botta G (1999) PCR-RFLP-detected human papilloma virus infection in a group of Senegalese women attending an STD clinic and identification of a new HPV-68 subtype. Intervirology 42: 221-227.

6. Bayo S, Bosch FX, De Sanjosé S, Munoz N, Combita AL, et al. (2002) Risk factors of invasive cervical cancer in Mali. International journal of epidemiology 31: 202-209.

7. Bekele A, Baay M, Mekonnen Z, Suleman S, Chatterjee S (2010) Human papillomavirus type distribution among women with cervical pathology- study over 4 years at Jimma Hospital, southwest Ethiopia. Tropical Medicine & International Health 15: 890-893.

8. Blossom D, Beigi R, Farrell J, Mackay W, Qadadri B, et al. (2007) Human papillomavirus genotypes associated with cervical cytologic abnormalities and HIV infection in Ugandan women. J Med Virol 79: 758-765.

9. Bosch FX, Manos MM, MuN" d N, Sherman M, Jansen AM, et al. (1995) Prevalence of human papillomavirus in cervical cancer: a worldwide perspective. Journal of the National Cancer Institute 87: 796-802.

10. Castellsagué X, Klaustermeier J, Carrilho C, Albero G, Sacarlal J, et al. (2008) Vaccine‐related HPV genotypes in women with and without cervical cancer in Mozambique: Burden and potential for prevention. International Journal of Cancer 122: 1901-1904.

11. Chabaud M, Le Cann P, Mayelo V, Leboulleux D, Diallo A, et al. (1996) Detection by PCR of human papillomavirus genotypes in cervical lesions of Senegalese women. Journal of medical virology 49: 259-263.

12. Chaouki N, Bosch FX, Muñoz N, Meijer CJ, El Gueddari B, et al. (1998) The viral origin of cervical cancer in Rabat, Morocco. International journal of cancer 75: 546-554.

13. Cooper K, Herrington C, Graham A, Evans M, McGee J (1991) In situ evidence for HPV 16, 18, 33 integration in cervical squamous cell cancer in Britain and South Africa. Journal of clinical pathology 44: 406-409.

14. Cooper K, Herrington C, Graham A, Evans M, McGee J (1991) In situ human papillomavirus (HPV) genotyping of cervical intraepithelial neoplasia in South African and British patients: evidence for putative HPV integration in vivo. Journal of clinical pathology 44: 400-405.

15. Czegledy J, Rogo K, Evander M, Wadell G (1992) High-risk human papillomavirus types in cytologically normal cervical scrapes from Kenya. Medical microbiology and immunology 180: 321-326.

16. Dartell M, Rasch V, Kahesa C, Mwaiselage J, Ngoma T, et al. (2012) Human Papillomavirus Prevalence and Type Distribution in 3603 HIV-Positive and HIV-Negative Women in the General Population of Tanzania: The PROTECT Study. Sexually Transmitted Diseases 39: 201-208.

17. De Vuyst H, Steyaert S, Van Renterghem L, Claeys P, Muchiri L, et al. (2003) Distribution of human papillomavirus in a family planning population in Nairobi, Kenya. Sexually transmitted diseases 30: 137.

18. De Vuyst H, Lillo F, Broutet N, Smith JS (2008) HIV, human papillomavirus, and cervical neoplasia and cancer in the era of highly active antiretroviral therapy. European Journal of Cancer Prevention 17: 545.

19. De Vuyst H, Parisi MR, Karani A, Mandaliya K, Muchiri L, et al. (2010) The prevalence of human papillomavirus infection in Mombasa, Kenya. Cancer Causes and Control 21: 2309-2313.

20. De Vuyst H, Ndirangu G, Moodley M, Tenet V, Estambale B, et al. (2011) Prevalence of human papillomavirus in women with invasive cervical carcinoma by HIV status in Kenya and South Africa. International journal of cancer.

21. De Vuyst H, Ndirangu G, Moodley M, Tenet V, Estambale B, et al. (2012) Human papillomavirus prevalence in invasive cervical carcinoma by HIV Status. Infectious Agents and Cancer 7.

22. Denny L, Boa R, Williamson AL, Allan B, Hardie D, et al. (2008) Human papillomavirus infection and cervical disease in human immunodeficiency virus-1-infected women. Obstetrics & Gynecology 111: 1380.

23. Denny L, Adewole I, Anorlu R, Dreyer G, Moodley M, et al. (2014) Human papillomavirus prevalence and type distribution in invasive cervical cancer in sub‐Saharan Africa. International journal of cancer 134: 1389-1398.

24. Dols JA, Reid G, Brown JM, Tempelman H, Bontekoe TR, et al. (2012) HPV Type Distribution and Cervical Cytology among HIV-Positive Tanzanian and South African Women. ISRN Obstet Gynecol 2012: 514146.

25. Fanta B (2005) The distribution of Human Papilloma Virus infection in women with cervical histological abnormalities from an area with high incidence of cervical cancer. Ethiopian medical journal 43: 151.

26. Firnhaber C, Zungu K, Levin S, Michelow P, Montaner LJ, et al. (2009) Diverse and high prevalence of human papillomavirus associated with a significant high rate of cervical dysplasia in human immunodeficiency virus-infected women in Johannesburg, South Africa. Acta cytologica 53: 10-17.

27. Firnhaber C, Van Le H, Pettifor A, Schulze D, Michelow P, et al. (2010) Association between cervical dysplasia and human papillomavirus in HIV seropositive women from Johannesburg South Africa. Cancer Causes and Control 21: 433-443.

28. Gage JC, Ajenifuja KO, Wentzensen NA, Adepiti AC, Eklund C, et al. (2012) The age-specific prevalence of human papillomavirus and risk of cytologic abnormalities in rural Nigeria: Implications for screen-and-treat strategies. International Journal of Cancer 130: 2111-2117.

29. Garcia-Espinosa B, Nieto-Bona MP, Rueda S, Silva-Senchez LF, Piernas-Morales MC, et al. (2009) Genotype distribution of cervical human papillomavirus DNA in women with cervical lesions in Bioko, Equatorial Guinea. Diagnostic pathology 4: 31.

30. Gravitt PE, Kamath AM, Gaffikin L, Chirenje ZM, Womack S, et al. (2002) Human papillomavirus genotype prevalence in high-grade squamous intraepithelial lesions and colposcopically normal women from Zimbabwe. International journal of cancer 100: 729-732.

31. Hammouda D, Munoz N, Herrero R, Arslan A, Bouhadef A, et al. (2005) Cervical carcinoma in Algiers, Algeria: human papillomavirus and lifestyle risk factors. International journal of cancer 113: 483-489.

32. Hammouda D, Clifford GM, Pallardy S, Ayyach G, Chékiri A, et al. (2011) Human papillomavirus infection in a population‐based sample of women in Algiers, Algeria. International journal of cancer 128: 2224-2229.

33. Icenogle JP, Laga M, Miller D, Manoka AT, Tucker RA, et al. (1992) Genotypes and Sequence Variants of Human Papillomavirus DNAs from Human Immunodeficiency Virus Type I-Infected Women with Cervical Intraepithelial Neoplasia. Journal of Infectious Diseases 166: 1210-1216.

34. Jaquet A, Horo A, Charbonneau V, Ekouevi DK, Roncin L, et al. (2012) Cervical human papillomavirus and HIV infection in women of child-bearing age in Abidjan, Cote d'Ivoire, 2010. Br J Cancer 107: 556-563.

35. Jones HE, Allan BR, van de Wijgert JHHM, Altini L, Taylor SM, et al. (2007) Agreement between self-and clinician-collected specimen results for detection and typing of high-risk human papillomavirus in specimens from women in Gugulethu, South Africa. Journal of clinical microbiology 45: 1679-1683.

36. Kay P, Soeters R, Nevin J, Denny L, Dehaeck C, et al. (2003) High prevalence of HPV 16 in South African women with cancer of the cervix and cervical intraepithelial neoplasia. Journal of medical virology 71: 265-273.

37. Keita N, Clifford G, Koulibaly M, Douno K, Kabba I, et al. (2009) HPV infection in women with and without cervical cancer in Conakry, Guinea. British Journal of Cancer 101: 202-208.

38. La Ruche G, You B, Mensah-Ado I, Bergeron C, Montcho C, et al. (1998) Human papillomavirus and human immunodeficiency virus infections: relation with cervical dysplasia-neoplasia in African women. International journal of cancer 76: 480-486.

39. MacLeod IJ, O'Donnell B, Moyo S, Lockman S, Shapiro RL, et al. (2011) Prevalence of human papillomavirus genotypes and associated cervical squamous intraepithelial lesions in HIV‚Äêinfected women in Botswana. Journal of medical virology 83: 1689-1695.

40. Marais DJ, Rose RC, Lane C, Kay P, Nevin J, et al. (2000) Seroresponses to human papillomavirus types 16, 18, 31, 33, and 45 virus-like particles in South African women with cervical cancer and cervical intraepithelial neoplasia. Journal of medical virology 60: 403-410.

41. Marais DJ, Passmore JAS, Denny L, Sampson C, Allan BR, et al. (2008) Cervical and oral human papillomavirus types in HIV-1 positive and negative women with cervical disease in South Africa. Journal of medical virology 80: 953-959.

42. Maranga IO, Hampson L, Oliver AW, He X, Gichangi P, et al. (2013) HIV Infection Alters the Spectrum of HPV Subtypes Found in Cervical Smears and Carcinomas from Kenyan Women. Open Virol J 7: 19-27.

43. Mayaud P, Weiss HA, Lacey CJ, Gill DK, Mabey DC (2003) Genital human papillomavirus genotypes in northwestern Tanzania. J Clin Microbiol 41: 4451-4453.

44. McDonald AC, Denny L, Wang C, Tsai WY, Wright TC, Jr., et al. (2012) Distribution of high-risk human papillomavirus genotypes among HIV-negative women with and without cervical intraepithelial neoplasia in South Africa. PLoS One 7: e44332.

45. Mzibri ME, Mhand RA, Benider A, Benchekroun N, Benchekroun M, et al. (2009) Molecular detection and genotyping of human papillomavirus in cervical carcinoma biopsies in an area of high incidence of cancer from Moroccan women. Journal of medical virology 81: 678-684.

46. Moodley J, Constant D, Hoffman M, Salimo A, Allan B (2009) Human papillomavirus prevalence, viral load and pre-cancerous lesions of the cervix in women initiating highly active antiretroviral therapy in South Africa: a cross-sectional study. BMC cancer 9: 275.

47. Naucler P, Da Costa FM, Ljungberg O, Bugalho A, Dillner J (2004) Human papillomavirus genotypes in cervical cancers in Mozambique. Journal of general virology 85: 2189-2190.

48. Naucler P, Mabota da Costa F, da Costa JL, Ljungberg O, Bugalho A, et al. (2011) Human papillomavirus type-specific risk of cervical cancer in a population with high human immunodeficiency virus prevalence: case-control study. J Gen Virol 92: 2784-2791.

49. Ndiaye C, Alemany L, Ndiaye N, Kamate B, Diop Y, et al. (2012) Human papillomavirus distribution in invasive cervical carcinoma in sub-Saharan Africa: could HIV explain the differences? Trop Med Int Health.

50. Ng'andwe C, Lowe JJ, Richards PJ, Hause L, Wood C, et al. (2007) The distribution of sexually-transmitted Human Papillomaviruses in HIV positive and negative patients in Zambia, Africa. BMC infectious diseases 7: 77.

51. Odida M, de Sanjosé S, Quint W, Bosch XF, Klaustermeier J, et al. (2008) Human Papillomavirus type distribution in invasive cervical cancer in Uganda. Bmc Infectious Diseases 8: 85.

52. Odida M, de Sanjose S, Sandin S, Quiros B, Alemany L, et al. (2010) Comparison of human papillomavirus detection between freshly frozen tissue and paraffin embedded tissue of invasive cervical cancer. Infect Agent Cancer 5: 15.

53. Odida M, Sandin S, Mirembe F, Kleter B, Quint W, et al. (2011) HPV types, HIV and invasive cervical carcinoma risk in Kampala, Uganda: a case-control study. Infectious Agents and Cancer 6: 8.

54. Okolo C, Franceschi S, Adewole I, Thomas JO, Follen M, et al. (2010) Human papillomavirus infection in women with and without cervical cancer in Ibadan, Nigeria. Infectious Agents and Cancer 5: 24.

55. Piras F, Piga M, De Montis A, Zannou ARF, Minerba L, et al. (2011) Prevalence of human papillomavirus infection in women in Benin, West Africa. Virology Journal 8: 514.

56. Rahman M, Sasagawa T, Yamada R, Kingoro A, Ichimura H, et al. (2011) High prevalence of intermediate‐risk human papillomavirus infection in uterine cervices of kenyan women infected with human immunodeficiency virus. J Med Virol 83: 1988-1996.

57. Ramesar JE, Dehaeck CMC, Soeters R, Williamson A (1996) Human papillomavirus in normal cervical smears from Cape Town. SOUTH AFRICAN MEDICAL JOURNAL-CAPE TOWN-MEDICAL ASSOCIATION OF SOUTH AFRICA- 86: 1402-1405.

58. Ramogola-Masire D, de Klerk R, Monare B, Ratshaa B, Friedman HM, et al. (2012) Cervical Cancer Prevention in HIV-Infected Women Using the "See and Treat" Approach in Botswana. Jaids-Journal of Acquired Immune Deficiency Syndromes 59: 308-313.

59. Richter KL, Van Rensburg EJ, Van Heerden WFP, Boy SC (2008) Human papilloma virus types in the oral and cervical mucosa of HIV-positive South African women prior to antiretroviral therapy. Journal of oral pathology & medicine 37: 555-559.

60. Rogo K (1990) Human papillomavirus and human immunodeficiency virus infection in relation to cervical cancer: Studies and observations of basic clinical and epidemiological aspects of cancer of the cervix with special reference to Kenya, Africa: Umea University.

61. Sahasrabuddhe V, Mwanahamuntu M, Vermund S, Huh W, Lyon M, et al. (2007) Prevalence and distribution of HPV genotypes among HIV-infected women in Zambia. British Journal of Cancer 96: 1480-1483.

62. Said H, Ahmed K, Burnett R, Allan B, Williamson AL, et al. (2009) HPV genotypes in women with squamous intraepithelial lesions and normal cervixes participating in a community-based microbicide study in Pretoria, South Africa. Journal of Clinical Virology 44: 318-321.

63. Singh DK, Anastos K, Hoover DR, Burk RD, Shi Q, et al. (2009) Human Papillomavirus Infection and Cervical Cytology in HIV-Infected and HIV-Uninfected Rwandan Women. Journal of Infectious Diseases 199: 1851-1861.

64. Stanczuk GA, Kay P, Allan B, Chirara M, Tswana SA, et al. (2003) Detection of human papillomavirus in urine and cervical swabs from patients with invasive cervical cancer. Journal of medical virology 71: 110-114.

65. Stanczuk GA, Kay P, Sibanda E, Allan B, Chirara M, et al. (2003) Typing of human papillomavirus in Zimbabwean patients with invasive cancer of the uterine cervix. Acta obstetricia et gynecologica Scandinavica 82: 762-766.

66. Thomas J, Herrero R, Omigbodun A, Ojemakinde K, Ajayi I, et al. (2004) Prevalence of papillomavirus infection in women in Ibadan, Nigeria: a population-based study. British Journal of Cancer 90: 638-645.

67. Vidal AC, Murphy SK, Hernandez BY, Vasquez B, Bartlett JA, et al. (2011) Distribution of HPV genotypes in cervical intraepithelial lesions and cervical cancer in Tanzanian women. Infectious Agents and Cancer 6: 1-8.

68. Wall S, Scherf C, Morison L, Hart K, West B, et al. (2005) Cervical human papillomavirus infection and squamous intraepithelial lesions in rural Gambia, West Africa: viral sequence analysis and epidemiology. British Journal of Cancer 93: 1068-1076.

69. Williamson AL, Dehaeck C, Soeters R (1989) Typing of human papillomaviruses in cervical intraepithelial neoplasia grade 3 biopsies from Cape Town. Journal of medical virology 28: 146-149.

70. Williamson AL, Brink NS, Dehaeck C, Ovens S, Soeters R, et al. (1994) Typing of human papillomaviruses in cervical carcinoma biopsies from Cape Town. Journal of medical virology 43: 231-237.

71. Xi LF, Touré P, Critchlow CW, Hawes SE, Dembele B, et al. (2003) Prevalence of specific types of human papillomavirus and cervical squamous intraepithelial lesions in consecutive, previously unscreened, West-African women over 35 years of age. International journal of cancer 103: 803-809.
