# Supplementary material for: Prevalence of Human Papillomavirus Genotypes among African Women with Normal Cervical Cytology and Neoplasia: A Systematic Review and Meta-Analysis
Source: PLoS One. 2015 Apr 14;10(4):e0122488. doi: 10.1371/journal.pone.0122488 (PMC4396854; doi:10.1371/journal.pone.0122488)
Supplement: S3 Table — (DOCX) [file pone.0122488.s005.docx]

|  | | **Table S3.** Overall statistics summary for any HPV prevalence categorized by source of tissues, countries, and GNI classification according to World Bank | | | | | | | | | |
| --- | --- | --- | --- | --- | --- | --- | --- | --- | --- | --- | --- |
|  |  | **ICC** | | **HSIL** | | **LSIL** | | **ASCUS** | | **Normal** | |
|  |  | **Cases (Studies)** | **% Prevalence (95% CI)** | **Cases (Studies)** | **% Prevalence (95% CI)** | **Cases (Studies)** | **% Prevalence (95% CI)** | **Cases (Studies)** | **% Prevalence (95% CI)** | **Cases (Studies)** | **% Prevalence (95% CI)** |
| TISSUE SOURCE | Exfoliated | 1528 (19) | 92.9 (90.0-95.7) | 1138 (27) | 89.6 (85.7-93.5) | 1028 (23) | 74.6 (66.5-82.6 | 333 (13) | 63.1 (42.4-83.8) | 4455 (39) | 40.9 (33.8-48.2) |
|  | Fixed | 1323 (13) | 88.0 (83.2-92.8) | 252 (5) | 81.3 (61.7) | 59 (2) | 88.2 (66.1-110.4) | 141 (2) | 65.7 (18.1-133.2) | 482 (2) | 28.1 (-5.4-61.5) |
|  | Fresh-frozen | 860 (11) | 91.4 (88.4-94.5) | 227 (2) | 78.2 (57.6-98.8) | . | . | . | . | 95 (1) | 30.7 (25.2-36.0) |
|  | Not specified | 30 (1) | 75.0 (60.7-89.3) | . | . | . | . | . | . | 42 (1) | 44.2 (33.8-54.6) |
| COUNTRY | Algeria | 176 (2) | 90.1 (69.2-111.1) |  |  |  |  |  |  | 57 (2) | 8.3 (1.5-15.2) |
|  | Benin | 5 (1) | 83.3 (51.5-115.2) | 3 (1) | 75.0 (35-115) | 5 (1) | 71.4 (37.8-105.1) | 33 (1) | 45.8 (33.8-57.8) | 69 (1) | 26.7 (21.2-32.3) |
|  | Botswana |  |  | 111 (2) | 85.8 (62.1-109.5) | 40 (1) | 74.074 (61.7-86.4) | 9 (1) | 75.0 (49.2-100.9) | 20 (1) | 50.0 (33.8-66.2) |
|  | Burkina Faso |  |  |  |  |  |  | 9 (1) | 100.0 (83.2-116.8) |  |  |
|  | Cote d'Ivoire | 125 (2) | 74.3 (39.9-108.8) | 49 (1) | 81.7 (71.2-92.1) | 103 (1) | 68.2 (60.5-75.9) |  |  | 375 (2) | 43.6 (5.8-81.5) |
|  | Eqt Guinea | 3 (1) | 100.0 (64.6-135.4) | 12 (1) | 70.6 (47.8-93.4) | 0 (1) | 0.0 (-42.1-42.1) |  |  |  |  |
|  | Ethiopia | 284 (2) | 95.2 (91.1-99.3) | 41 (1) | 87.4 (76.8-97.9) | 11 (1) | 100.0 (85.8-114.2) | 71 (1) | 89.9 (82.6-97.1) | 4 (1) | 50.0 (15.7-84.3) |
|  | Gambia | 1 (1) | 100.0 (51.3-148.8) | 13 (1) | 86.7 (67.3-106.1) |  |  |  |  |  |  |
|  | Ghana | 153 (1) | 93.9 (89.9-97.9) |  |  |  |  |  |  |  |  |
|  | Guinea | 88 (2) | 95.0 (86.1-103.8) |  |  |  |  |  |  | 360 (1) | 47.8 (44.2-51.5) |
|  | Kenya | 268 (3) | 90.3 (74.7-105.9) | 141 (3) | 82.0 (64.9-99.1) | 206 (3) | 57.4 (46.3-68.5) | 60 (1) | 49.2 (40.0-58.4) | 545 (5) | 41.9 (31.7-52.2) |
|  | Mali | 143 (3) | 93.1 (87.1-99.0) |  |  |  |  |  |  | 4 (1) | 33.3 (5.7-60.9) |
|  | Morocco | 218 (2) | 88.9 (82.3-95.6) | 3 (1) | 42.9 (7.0-78.7) | 4 (1) | 16.7 (0.3-33.0) | 3 (1) | 6.5 (-1.7-14.8) | 162 (2) | 16.3 (14.0-18.7) |
|  | Mozambique | 486 (30 | 94.0 (85.2-102.9) |  |  |  |  |  |  | 211 (2) | 53.5 (9.5-97.4) |
|  | Nigeria | 220 (2) | 87.3 (81.7-92.8) |  |  |  |  |  |  | 322 (2) | 17.6 (3.6-31.6) |
|  | Rwanda |  |  |  |  | 86 (1) | 86.9 (79.8-94.0) | 34 (1) | 77.3 (64.1-90.5) | 260 (1) | 59.8 (55.1-64.5) |
|  | Senegal | 110 (1) | 83.3 (76.6-90.0) | 22 (1) | 95.7 (84.7-106.6) | 83 (2) | 72.1 (31.3-113.0) | 52 (1) | 20.5 (15.3-25.6) | 233 (3) | 12.7 (11.1-14.3) |
|  | South Africa | 388 (6) | 83.1 (73.6-92.6) | 585 (12) | 86.9 (79.5-94.5) | 439 (8) | 90.9 (84.2-97.6) | 117 (6) | 76.1 (50.6-101.5) | 1512 (11) | 57.9 (39.6-76.3) |
|  | Sudan | 79 (1) | 94.1 (88.4-99.7) | 1 (1) | 100.0 (51.3-148.8) |  |  |  |  |  |  |
|  | Tanzania | 81 (3) | 83.4 (67.4-99.5) | 147 (3) | 93.8 (89.6-98.0) | 70 (3) | 79.3 (58.4-100.3) | 70 (1) | 41.4 (33.8-49.1) | 669 (3) | 19.9 (8.7-31.2) |
|  | Uganda | 520 (4) | 84.1 (72.6-95.5) | 14 (1) | 73.7 (52.7-94.7) |  |  |  |  | 95 (1) | 30.7 (25.5-36.0) |
|  | Zaire (DRC) |  |  | 2 (2) | 100.0 (57.9-142.1) | 6 (1) | 75.0 (44.1-106.0) |  |  |  |  |
|  | Zambia | 28 (1) | 100.0 (93.8-106.2) | 49 (1) | 100.0 (96.4-103.6) | 34 (1) | 97.1 (89.7-104.6) | 25 (1) | 96.2 (86.4-105.9) | 54 (2) | 68.0 (57.4-78.7) |
|  | Zimbabwe | 140 (2) | 99.8 (98.0-101.6) | 162 (1) | 77.1 (71.3-83.0) |  |  |  |  | 64 (1) | 30.1 (23.7-36.4) |
|  | Mixed5 | 225 (1) | 95.8 (92.9-98.6) | 16 (1) | 94.1 (79.8-108.4) |  |  |  |  | 58 (1) | 42.0 (33.5-50.5) |
| GNI | Low income | 2016 (24) | 91.4 (88.5-94.4) | 620 (13) | 86.9 (80.9-92.8) | 384 (10) | 74.3 (62.3-86.3) | 268 (5) | 60.7 (39.4-82.0) | 2327 (18) | 39.8 (30.3-49.2) |
|  | Lower-middle income | 933 (10) | 90.0 (86.2-93.7) | 140 (5) | 88.8 (76.1-101.4) | 224 (5) | 65.9 (42.9-89.0) | 80 (3) | 40.9 (-4.9-86.7) | 1100 (10) | 25.8 (17.6-34.1) |
|  | Upper-middle income | 564 (8) | 85.6 (78.3-92.8) | 823 (14) | 87.0 (80.7-93.4) | 479 (9) | 88.8 (82.1-95.7) | 126 (7) | 75.9 (53.0-99.0) | 1589 (14) | 50.3 (32.8-67.9) |
|  | High income | 3 (1) | 100.0 (64.6-135.4) | 17 (1) | 70.6 (47.8-93.4) | 0 (1) | 0.0 (-42.1-42.1) | . | . | . | . |
|  | Mixed economies | 225 (1) | 95.8 (92.9-98.6) | 17 (1) | 94.1 (79.8-108.4) |  | . | . | . | 58 (1) | 42.0 (33.5-50.5) |
|  | **Total** | **3741 (43)** | **90.5 (88.2-92.7)** | **1617 (33)** | **87.2 (83.3-91.1)** | **1087 (23)** | **75.8 (68.3-83.4)** | **474 (15)** | **63.5 (45.3-81.6)** | **5074 (43)** | **40.2 (33.9-46.5)** |

**Abbreviations**: Lesions-ASCUS: Atypical squamous cells of undetermined significance; LSIL: Low-grade squamous intraepithelial lesions; HSIL: High-grade squamous intraepithelial lesions; ICC: Invasive cervical cancer (this included SCC and ADC). CI: Confidence interval; N: Number of cases tested for a given HPV type. Blank boxes means indeterminate.
