# Supplementary material for: Prevalence of Human Papillomavirus Genotypes among African Women with Normal Cervical Cytology and Neoplasia: A Systematic Review and Meta-Analysis
Source: PLoS One. 2015 Apr 14;10(4):e0122488. doi: 10.1371/journal.pone.0122488 (PMC4396854; doi:10.1371/journal.pone.0122488)
Supplement: S4 Table — (PDF) [file pone.0122488.s006.pdf]

Country (S)<br>N [%]<br>{Region}<br>GNI	Study	HIV (Y/N)	Design	Median Age	Age range	Pathology	Primer	HPV genotyping location	Cervical Lesion/Norm	N	Any	HPV-specific prevalence (% of all cases tested)																		
												16	18	31	33	35	39	45	51	52	53	56	58	59	66	68	6	11	Other	Multi
**Algeria** (3)<br>1087 [4.1]<br>{Northern Africa}<br>Upper Middle Income	Bosch 1995a	N	C-S	34	.	H	PGMY9/11	USA	ICC	12	75	33.3	41.7	0	.	.	0	0	.	.	.	.	.	0	.	.	0	0	0	.
	Hammouda 2005	N	C-C	52	30-88	C	GP5/6	NLD	Norm	145	12.4	7.6	2.1	1.4	0	0.7	0.7	0.7	0	0	0	0	0	0	0	0	0	0	2.8	4.8
	Hammouda 2005	N	C-C	53	32-82	C	GP5/6		ICC	171	97.7	57.9	14.6	2.3	4.7	4.1	2.3	6.4	1.8	0.6	0	1.8	0.6	1.2	0	0	0	0	1.8	9.4
	Hammouda 2011	N	C-S	39.9	15-65	C	GP5/6		Norm	732	5.3	0.5	0.3	1.1	0.1	0.1	0.1	0.3	0.4	0.1	0	0.1	0	0.1	0	0	0.5	0	1.6	1.1
	Hammouda 2011	N	C-S	39.9	15-65	H	GP5/6		ASC+	27	33.3	3.7	0	7.4	3.7	0	3.7	0	3.7	0	3.7	7.4	0	0	0	0	0	0	3.7	14.8
**Benin** (2)<br>360 [1.3]<br>{Western Africa}<br>Low Income	Bosch 1995b	N	C-S	34	.	H	PGMY9/11	USA	ICC	6	83.3	50	16.7	0	.	.	0	0	.	.	.	.	.	0	.	.	0	0	0	.
	Piras 2011	N	C-S	.	15-70	C	GP5/6	ITA	Norm	258	26.7	4.3	3.1	0.8	0.8	4.7	0.4	1.6	1.2	0.4	0	2.3	3.1	8.5	0	0	2.3	8.3	4.7	9.3
	Piras 2011	N	C-S	.	15-70	C	GP5/6		ASC	72	45.8	8.3	6.9	1.4	4.2	12.5	0	9.7	1.4	1.4	0	6.9	4.2	9.7	0	0	1.4	14.3	6.9	18.1
	Piras 2011	N	C-S	.	15-70	C	GP5/6		LSIL	7	71.4	14.3	0	0	0	0	0	0	14.3	0	0	0	14.3	14.3	0	0	42.9	75	14.3	71.4
	Piras 2011	N	C-S	.	15-70	C	GP5/6		HSIL	4	75	0	0	0	0	25	0	0	0	25	0	0	0	0	0	0	0	0	0	0
	Piras 2011	N	C-S	.	15-70	C	GP5/6		HSIL+	7	57.1	0	0	14.3	0	14.3	0	14.3	0	0	0	0	0	14.3	0	0	0	0	0	28.6
	Piras 2011	N	C-S	.	15-70	C	GP5/6		UNS	6	83.3	16.7	33.3	16.7	16.7	0	0	0	0	0	0	0	0	0	0	0	0	0	0	33.3
**Botswana** (2)<br>227 [0.9]<br>{Southern Africa}<br>Upper Middle Income	MacLeod 2011	Y	C-S	32	29-39	C	PGMY9/11	USA	Norm	40	50	12.5	5	0	2.5	5	7.5	2.5	2.5	5	12.5	2.5	12.5	15	0	5	0	0	17.5	30
	MacLeod 2011	Y	C-S	32	29-39	C	PGMY9/11		ASC	12	75	0	16.7	8.3	8.3	0	8.3	8.3	0	8.3	0	0	25	0	8.3	0	0	3.7	16.7	50
	MacLeod 2011	Y	C-S	32	29-39	C	PGMY9/11		LSIL	54	74.1	13	9.3	3.7	0	5.6	14.8	9.3	7.4	9.3	11.1	1.9	18.5	13	18.5	1.9	1.9	4.8	48.1	53.7
	MacLeod 2011	Y	C-S	32	29-39	C	PGMY9/11	ZFA	HSIL	21	71.4	23.8	23.8	0	4.8	14.3	23.8	14.3	0	23.8	4.8	0	23.8	14.3	9.5	14.3	0	0	38.1	57.1
	Ramogola-Masire 2011	Y	C-S	36	>21	H	PGMY9/11	USA	HSIL	100	96	45	27	14	18	40	3	13	15	9	16	10	35	8	20	12	8	0.6	210	82
**Cote d'Ivoire** (3)<br>1192 [4.5]<br>{Western Africa}<br>Low Middle Income	Adjorlolo-Johnson 2010	Y/N	C-C	46.7	24-69	C	PGMY9/11		ICC	132	89.4	44.7	21.2	3	.	8.3	.	9.8	.	.	.	.	.	.	.	.	.	.	0	.
	Jaquet 2012	Y	C-S	.	25-65	C	PGMY9/11	FRA	Norm	445	62.9	12.4	9.2	4.3	2.7	13.3	3.4	5.4	5.6	4.7	8.1	3.1	8.3	6.7	1.8	1.8	5.2	0.3	64.5	12.8
	La Ruche 1998	Y/N	C-S	30	20-50	C	MY9/11	FRA	Norm	391	24.3	1.5	0.3	0.8	0	0.5	0.3	0.3	0	0.3	0	0	1	0.3	0	0.8	0.5	0.7	1.5	2.3
	La Ruche 1998	Y/N	C-S	27.9	20-50	C	MY9/11		LSIL	151	68.2	10.6	6	5.3	5.3	1.3	2	2	0	2.6	0	2.6	3.3	1.3	0	2.6	3.3	1.7	14.6	23.2
	La Ruche 1998	Y/N	C-S	30	20-50	C	MY9/11		HSIL	60	81.7	25	8.3	5	6.7	0	0	0	0	3.3	0	3.3	6.7	0	0	1.7	0	0	15	23.3
	La Ruche 1998	Y/N	C-S	41.8	20-50	C	MY9/11		ICC	13	53.8	38.5	0	0	0	0	0	0	0	7.7	0	0	7.7	0	0	0	0	0	0	0

Country (S) N [%] {Region} GNI	Study	HIV (Y/N)	Design	Median Age	Age range	Pathology	Primer	HPV genotyping location	Cervical Lesion/Norm	N	Any	16	18	31	33	35	39	45	51	52	53	56	58	59	66	68	6	11	Other	Multi
												HPV-specific prevalence (% of all cases tested)																		
**Equatorial Guinea** (1) 22 [0.1] {Middle Africa} High Income	Garcia-Espinosa 2009	N	C-S	.	15-71	C	PGMY9/11	ESP	LSIL	2	0	0	0	0	0	0	0	0	0	0	0	0	0	0	0	0	0	0	0	0
	Garcia-Espinosa 2009	N	C-S	.	15-71	C	PGMY9/11		HSIL	17	70.6	17.6	5.9	5.9	17.6	0	0	0	0	5.9	0	0	11.8	0	0	0	0	0	0	5.9
	Garcia-Espinosa 2009	N	C-S	.	15-71	C	PGMY9/11		ICC	3	100	33.3	0	0	33.3	0	0	0	0	0	0	0	33.3	0	0	0	0	0	0	0
**Ethiopia** (3) 566 [2.1] {Eastern Africa} Low Income	Abate 2013a	N	C-S	48	30-75	H	SPF	ETH	LSIL	11	100	100	27.3	18.2	18.2	0	0	9.1	0	18.2	0	0	27.3	0	0	0	0	0	0	.
	Abate 2013a	N	C-S	48	30-75	H	SPF		HSIL	13	84.6	61.5	15.4	0	7.7	0	0	7.7	0	38.5	0	0	15.4	0	0	0	7.7	0	0	0
	Abate 2013a	N	C-S	48	30-75	H	SPF		ICC	136	92.6	86	19.1	5.9	5.9	4.4	3.7	11.8	0	22.8	0	0.7	20.6	0	1.5	6.6	10.3	0	0	3.7
	Bekele 2010	N	C-S	45	32-65	H	GP5/6		HSIL+	122	67.2	55.7	8.2	1.6	0.8	1.6	3.3	4.1	0	1.6	0	8.2	0.8	0.8	0	0	.	.	0	36.9
	Fanta 2005	N	C-S	38	.	H	GP5/6		Norm	8	50	0	0	12.5	0	0	0	0	0	0	0	0	0	0	0	0	0	0	0	25
	Fanta 2005	N	C-S	38	.	H	GP5/6		ASC	79	89.9	77.2	5.1	0	0	0	0	0	0	0	1.3	0	1.3	0	0	0	0	0	0	3.8
	Fanta 2005	N	C-S	38	.	H	GP5/6		HSIL	34	88.2	64.7	5.9	2.9	2.9	0	0	0	0	0	0	0	0	0	2.9	0	0	0	0	88.2
	Fanta 2005	N	C-S	40	.	H	GP5/6		ICC	163	96.9	71.8	18.4	0	0	0	0	0	0	0	0	0	0.6	0	0	0	0	0	0	4.3
**Gambia** (1) 16 [0.1] {Western Africa} Low Income	Wall 2005	N	C-S	33.5	15-54	C	GP5/6	GBR	HSIL	15	86.7	20	6.7	6.7	20	6.7	6.7	6.7	0	0	0	0	20	0	0	0	0	0	0	0
	Wall 2005	N	C-S	33.5	15-54	C	GP5/6		ICC	1	100	100	0	0	0	0	0	0	0	0	0	0	0	0	0	0	0	0	0	0
**Ghana** (1) 163 [0.6] {Western Africa} Low Middle Income	Denny 2013a	Y/N	C-S	58	25-96	H	SPF	NLD	ICC	163	93.9	41.1	14.1	0.6	2.5	4.9	0.6	4.9	1.2	1.8	0	1.2	0.6	0	0	.	.	.	0.6	17.8
**Guinea** (2) 926 [3.5] {Western Africa} Low Income	Bosch 1995c	N	C-S	34	.	H	PGMY9/11	USA	ICC	18	100	38.9	5.6	22.2	.	.	0	33.3	.	.	.	.	.	0	.	.	0	0	0	.
	Keita 2009	N	C-S	45	18-64	C	GP5/6	NLD	Norm	752	47.9	6.6	3.2	1.3	2.9	3.2	0.9	4.7	1.9	4	0.8	2.1	3.2	0.8	3.5	0.9	1.2	3.9	19.8	15.8
	Keita 2009	N	C-S	45	18-64	H	GP5/6		ICC	77	90.9	48.1	13	0	2.6	3.9	2.6	16.9	0	0	0	0	0	0	0	0	0	0	2.6	3.9
	Keita 2009	N	C-S	45	18-64	H	GP5/6		ASC+	79	78.5	13.9	3.8	6.3	11.4	5.1	0	6.3	2.5	8.9	8.9	2.5	5.1	0	10.1	1.3	2.5	0	26.6	35.4

												HPV-specific prevalence (% of all cases tested)																		
Country (S)<br>N [%]<br>{Region}<br>GNI	Study	HIV (Y/N)	Design	Median Age	Age range	Pathology	Primer	HPV genotyping location	Cervical Lesion/Norm	N	Any	16	18	31	33	35	39	45	51	52	53	56	58	59	66	68	6	11	Other	Multi
**Kenya** (8)<br>2253 [8.4]<br>{Eastern Africa}<br>Low Income	Czegledy 1992	N	C-S	25.8	20-35	C	IH	SWE	Norm	77	19.5	6.5	3.9	0	1.3	.	.	.	.	.	.	.	.	.	.	.	.	.	9.1	1.3
	De Vuyst 2003	N	C-S	35	25-55	C	SPF	BEL	Norm	369	38.8	3.5	2.2	3.3	1.9	2.7	1.4	1.6	1.1	6.2	2.7	1.4	2.7	0	3.5	1.6	0.5	6.7	11.4	.
	De Vuyst 2003	N	C-S	35	25-55	C	SPF		LSIL	30	60	13.3	6.7	3.3	0	23.3	0	3.3	3.3	13.3	10	3.3	6.7	0	3.3	3.3	0	0	10	.
	De Vuyst 2003	N	C-S	35	25-55	C	SPF	BEL	HSIL	29	96.6	34.5	3.4	6.9	3.4	17.2	0	6.9	10.3	24.1	0	3.4	6.9	0	10.3	6.9	0	0	3.4	.
	De Vuyst 2008	Y/N	C-S	42.55		C	SPF	ITA	ICC	204	97.5	43.1	20.1	3.9	3.4	5.9	2.5	13.7	1	8.8	1	2	0.5	0.5	1.5	1.5	0	0.4	2.9	19.6
	De Vuyst 2010	N	C-S	.	>15	C	PGMY9/11	ITA	Norm	454	40.3	7.5	4.4	1.8	3.3	0.4	0.9	1.8	3.5	4.2	6.6	1.8	9.9	1.3	2.6	1.5	4.6	33.3	22	32.6
	De Vuyst 2010	N	C-S	.	>15	C	SPF		ASC+	42	59.5	9.5	7.1	2.4	0	2.4	2.4	7.1	0	2.4	7.1	16.7	16.7	0	2.4	2.4	2.4	0	16.7	28.6
	De Vuyst 2012	Y	C-S	38.1	18 - 55	H	GP5/6	BEL	Norm	172	60.5	2.9	5.2	5.2	0	9.9	2.3	4.1	2.3	7.6	0	6.4	1.7	0	0	2.9	2.9	0	2.3	32
	De Vuyst 2012	Y	C-S	38.1	18 - 55	H	GP5/6		LSIL	186	64	9.7	3.2	2.2	5.4	9.1	1.1	3.8	4.3	6.5	0	4.8	4.3	0.5	0	1.6	1.6	0.9	1.6	34.9
	De Vuyst 2012	Y	C-S	38.1	18 - 55	H	GP5/6		HSIL	113	90.3	26.5	10.6	10.6	4.4	19.5	7.1	9.7	6.2	9.7	0	8.8	12.4	3.5	0	1.8	5.3	1.3	0	63.7
	Maranga 2013	Y/N	C-S	42	27-68	C	IH	GBR	ICC	77	72.7	44.2	27.3	.	.	.	.	9.1	.	0	.	0	0	.	.	.	.	.	0	.
	Rahman 2011	Y/N	C-S	31.2	16-61	C	GP5/6	JPN	Norm	201	49.8	5.5	3.5	4.5	1	4.5	12.9	4.5	2.5	11.9	8.5	6	1.5	4.5	3.5	4	2.5	5.7	17.4	.
	Rahman 2011	Y/N	C-S	31.2	16-61	C	GP5/6		ASC	122	49.2	4.1	0	4.9	2.5	3.3	7.4	10.7	9.8	11.5	2.5	5.7	1.6	4.1	7.4	8.2	2.5	1.4	25.4	.
	Rahman 2011	Y/N	C-S	31.2	16-61	C	GP5/6		LSIL	141	48.9	5.7	5	8.5	5	2.8	5.7	8.5	3.5	7.8	6.4	9.9	3.5	3.5	4.3	3.5	2.1	4.3	24.1	.
	Rahman 2011	Y/N	C-S	31.2	16-61	C	GP5/6		HSIL	23	47.8	4.3	13	13	0	17.4	13	17.4	13	8.7	8.7	8.7	4.3	0	4.3	8.7	0	0	39.1	.
	Rogo 1990	N	C-S	34	28-51	C	IH	SWE	ICC	13	100	84.6	69.2	.	0	.	.	.	.	.	.	.	.	.	.	.	23.1	.	0	92.3
**Mali** (3)<br>167 [0.6]<br>{Western Africa}<br>Low Income	Bayo 2002	N	C-C	46.9	18-80	H	GP5/6	BEL	Norm	12	33.3	0	8.3	0	0	0	0	0	0	0	0	0	0	0	0	0	0	0	25	0
	Bayo 2002	N	C-C	46.9	18-80	H	GP5/6		ICC	65	96.9	47.7	12.3	0	1.5	1.5	0	10.8	6.2	0	0	0	3.1	0	0	0	0	0	0	10.8
	Bosch 1995d	N	C-S	34	.	H	PGMY9/11	USA	ICC	58	89.7	34.5	1.7	1.7	.	.	0	17.2	.	.	.	.	.	0	.	.	0	0	0	.
	Ndiaye 2012a	N	C-S	51.9	<39 - >60	H	SPF	ESP	ICC	32	87.5	40.6	6.3	.	.	.	.	12.5	.	.	.	.	.	.	.	.	0	.	0	0
**Morocco** (3)<br>1328 [5]<br>{Northern Africa}<br>Low Middle Income	Alhamany 2010	N	C-S	42.5	17-83	C	MY9/11	N.S.	Norm	785	15.8	2.2	1.4	0.5	0.1	0.4	.	0.3	.	.	.	.	.	.	.	.	.	.	11	0
	Alhamany 2010	N	C-S	42.5	17-80	C	MY9/11		ASC	46	6.5	2.2	2.2	2.2	2.2	0	.	0	.	.	.	.	.	.	.	.	.	.	0	2.2
	Alhamany 2010	N	C-S	42.5	17-82	C	MY9/11		LSIL	24	16.7	16.7	4.2	0	0	0	.	0	.	.	.	.	.	.	.	.	.	.	0	4.2
	Alhamany 2010	N	C-S	42.5	17-81	C	MY9/11		HSIL	7	42.9	14.3	28.6	28.6	0	0	.	0	.	.	.	.	.	.	.	.	.	.	0	14.3
	Alhamany 2010	N	C-S	42.5	17-80	C	MY9/11		ASC+	13	100	46.2	38.5	23.1	7.7	0	.	0	.	.	.	.	.	.	.	.	.	.	84.6	23.1
	Chaouki 1998	N	C-C	40.7	18-70	C	GP5/6	FRA	Norm	203	18.7	3.9	1	0	0	0.5	0	1.5	0.5	0.5	0	0.5	0.5	0	0	0.5	1	0	4.4	1
	Chaouki 1998	N	C-C	49.7	21-73	H	GP5/6		ICC	168	85.7	53	7.7	2.4	0.6	0.6	0	4.8	0	1.2	0	0	0	1.8	0.6	0	0.6	0	5.4	7.1
	Mzibri 2009	N	C-S	61	34-88	H	MY9/11	MAR	ICC	82	90.2	67.1	42.7	0	0	0	0	14.6	0	0	0	0	0	2.4	0	0	0	0	9.8	35.4
**Mozambique** (3)<br>970 [3.6]<br>{Eastern Africa}<br>Low Income	Castellsague 2008	N	C-S	34	14-61	C	SPF	ESP	Norm	195	75.9	6.2	10.8	10.3	5.6	14.9	9.2	3.1	17.9	9.7	4.6	3.6	1.5	0	6.2	7.2	5.1	3	20.5	37.9
	Castellsague 2008	N	C-S	34	14-61	C	SPF		ICC	230	100	47	31.3	2.6	4.8	10.4	1.3	12.6	15.2	14.3	1.7	0	1.7	0.4	0.4	3.5	1.3	8.2	7	35.2
	Castellsague 2008	N	C-S	34	14-61	C	SPF		ASC+	49	89.8	8.2	10.2	4.1	10.2	26.5	6.1	4.1	16.3	24.5	4.1	6.1	16.3	0	0	6.1	2	2.8	8.2	53.1
	Naucler 2004	Y/N	C-S	46		H	GP5/6	SWE	ICC	72	97.2	55.6	26.4	2.8	9.7	19.4	0	23.6	1.4	1.4	0	1.4	1.4	0	2.8	1.4	.	.	0	62.5

Country (S) N [%] {Region} GNI	Study	HIV (Y/N)	Design	Median Age	Age range	Pathology	Primer	HPV genotyping location	Cervical Lesion/Norm	N	Any	HPV-specific prevalence (% of all cases tested) 16	18	31	33	35	39	45	51	52	53	56	58	59	66	68	6	11	Other	Multi
	Naucler 2011	Y/N	C-C	45		H	GP5/6	SWE	Norm	203	31	6.4	3	2	1.5	1.5	1	3	0.5	5.4	.	1.5	3.9	0.5	4.4	0	0	0	8.9	9.4
	Naucler 2011	Y/N	C-C	33		H	GP5/6		ICC	221	84.2	49.8	20.4	0.9	3.2	3.6	0	14.5	1.4	2.3	.	1.8	0.9	2.3	1.8	0	0	0.7	0.9	19
**Nigeria** (4) 2485 [9.3] {Western Africa} Low Middle Income	Denny 2013c	Y/N	C-S	57	27-102	C	SPF	NLD	ICC	179	84.9	42.5	11.7	1.7	1.1	7.3	1.1	6.1	2.2	2.2	0.6	0.6	1.1	2.2	0	.	.	.	0	3.4
	Gage 2012	N	C-S	45	>15	C	MY9/11	USA	Norm	1075	10.5	1.5	1.2	2	0.2	1.5	0.3	0.4	0.8	1.9	0	0.6	1.7	0.4	0	0.7	.	.	0	2.2
	Gage 2012	N	C-S	45	>15	C	MY9/11		HSIL+	32	65.6	21.9	6.3	9.4	3.1	18.8	0	6.3	3.1	6.3	0	0	3.1	0	0	0	.	.	0	9.4
	Gage 2012	N	C-S	45	>15	C	MY9/11		ASC+	112	55.4	8.9	2.7	8	0.9	11.6	1.8	1.8	8.9	8	0	2.7	7.1	1.8	0	1.8	.	.	0	8
	Gage 2012	N	C-S	45	>15	C	MY9/11		UNS	80	51.3	3.8	1.3	7.5	0	8.8	2.5	0	11.3	8.8	0	3.8	8.8	2.5	0	2.5	.	.	0	7.5
	Okolo 2010	N	C-S	55	>15, 31-80	H	GP5/6	NLD	ICC	75	90.7	61.3	9.3	1.3	1.3	5.3	1.3	5.3	4	0	0	5.3	0	0	0	0	0	0	1.3	6.7
	Thomas 2004	N	C-S	45	>15	C	GP5/6	FRA	Norm	844	24.8	3	1.7	2.6	0.6	3	0.6	2.1	1.1	1.5	0.2	2.1	2.5	0.6	2	0.2	0.4	3.4	11.3	21.7
	Thomas 2004	N	C-S	45	>15	C	GP5/6		ASC+	88	40.9	5.7	4.5	5.7	0	5.7	1.1	1.1	5.7	4.5	0	4.5	2.3	0	3.4	0	5.7	0.6	19.3	15.9
**Rwanda** (1) 647 [2.4] {Eastern Africa} Low Income	Singh 2009	Y/N	C-S	34	>25	C	MY9/11	Not stated	Norm	435	59.8	6.2	4.1	2.8	2.5	4.6	1.1	1.4	5.1	5.1	6.4	2.1	5.3	1.6	1.1	3.7	2.8	9.1	25.3	27.1
	Singh 2009	Y/N	C-S	34	>25	C	MY9/11		ASC	44	77.3	2.3	9.1	2.3	6.8	6.8	0	0	4.5	6.8	4.5	4.5	9.1	2.3	2.3	6.8	4.5	0	15.9	29.5
	Singh 2009	Y/N	C-S	34	>25	C	MY9/11		LSIL	99	86.9	20.2	13.1	5.1	6.1	18.2	6.1	4	15.2	14.1	15.2	15.2	7.1	5.1	11.1	6.1	4	2.9	45.5	59.6
	Singh 2009	Y/N	C-S	34	>25	C	MY9/11		HSIL+	69	94.2	23.2	8.7	10.1	18.8	18.8	4.3	1.4	18.8	10.1	10.1	18.8	7.2	17.4	1.4	1.4	7.2	4.1	63.8	56.5
**Senegal** (4) 2492 [9.3] {Western Africa} Low Middle Income	Astori 1999	N	C-S	36	<30 - [illegible]	C	MY9/11	ITA	Norm	158	13.9	5.7	1.3	1.3	1.3	0	0	0	0	1.3	1.9	0	1.9	0	0	1.3	0	0	0	1.9
	Chabaud 1996	N	C-S	30	13-71	C	IH	FRA	Norm	26	23.1	19.2	3.8	0	0	.	.	.	.	.	.	.	.	.	.	.	0	0	0	.
	Chabaud 1996	N	C-S	30	13-71	C	IH		LSIL	42	92.9	40.5	33.3	2.4	0	0	.	11.9	.	4.8	.	.	0	.	.	0	28.6	26.1	9.5	.
	Chabaud 1996	N	C-S	30	13-71	C	IH		HSIL	23	95.7	43.5	47.8	0	4.3	0	0	8.7	.	0	.	.	0	.	.	4.3	21.7	8.7	0	.
	Chabaud 1996	N	C-S	30	13-71	C	IH		UNS	46	23.9	15.2	8.7	0	0	.	.	.	.	.	.	.	.	.	.	.	0	0	0	.
	Ndiaye 2012b	N	C-S	51.9	<39 - [illegible]	H	SPF		ICC	132	83.3	35.6	9.8	.	.	.	.	11.4	.	.	.	.	.	.	.	.	0	.	0	0
	Xi 2003	N	C-S	42.7	>35	C	MY9/11	USA	Norm	1639	12.5	1	0.9	0.4	0.7	0	0.1	0.2	0.3	0.5	0.7	0.3	0.7	0.4	0.2	0.1	0.2	0	7.4	1.2
	Xi 2003	N	C-S	42.7	>35	C	MY9/11		ASC	254	20.5	2.4	1.6	0.8	1.6	0.4	1.2	0.4	0	0.4	1.2	0.4	0.8	0	0.4	0	0	0	13.4	3.1
	Xi 2003	N	C-S	42.7	>35	C	MY9/11		LSIL	86	51.2	8.1	4.7	1.2	2.3	3.5	0	0	1.2	3.5	3.5	1.2	9.3	2.3	1.2	0	2.3	1.2	14	7
	Xi 2003	N	C-S	42.7	>35	C	MY9/11		HSIL+	86	75.6	23.3	4.7	5.8	8.1	1.2	1.2	0	3.5	8.1	2.3	1.2	12.8	0	2.3	1.2	2.3	1.4	22.1	15.1
**South Africa** (18) 5082 [19.0] {Southern Africa} Upper Middle Income	Allan 2008	N	C-S	44	21-59	C	PGMY9 /11	ZAF	Norm	848	20.4	2	1.3	0.5	1.1	0.6	0.6	1.4	0.7	1.9	2.2	0.4	1.1	1.1	0.2	1.3	0.5	1.7	5.7	8.8
	Allan 2008	N	C-S	44	21-59	C	PGMY9 /11		ASC	115	41.7	5.2	3.5	2.6	3.5	1.7	7	6.1	0.9	12.2	5.2	0.9	0.9	2.6	2.6	0.9	0.9	0	14.8	53
	Allan 2008	N	C-S	44	21-59	C	PGMY9 /11		LSIL	57	70.2	12.3	8.8	3.5	7	12.3	5.3	5.3	8.8	17.5	15.8	3.5	5.3	3.5	3.5	0	0	0	22.8	93
	Allan 2008	N	C-S	44	21-59	C	PGMY9 /11		HSIL	53	83	18.9	7.5	11.3	5.7	18.9	1.9	5.7	5.7	11.3	5.7	0	3.8	1.9	5.7	1.9	1.9	0	13.2	64.2
	Cooper 1991a	N	C-S	.	<30 - >60	H	IH	ZAF	HSIL	72	50	22.2	9.7	0	11.1	6.9	0	.	.	.	.	.	.	.	.	.	.	.	0	18.1

												HPV-specific prevalence (% of all cases tested)																		
Country (S) N [%] {Region} GNI	Study	HIV (Y/N)	Design	Median Age	Age range	Pathology	Primer	HPV genotyping location	Cervical Lesion/Norm	N	Any	16	18	31	33	35	39	45	51	52	53	56	58	59	66	68	6	11	Other	Multi
	Cooper 1991b	N	C-S	.	<30 - >60	H	IH	N.S.	ICC	69	63.8	42	21.7	0	0	0	0	.	.	.	.	.	.	.	.	.	.	.	0	.
	Denny 2008	Y/N	C-S	29.4	18-54	C	PGMY9 /11	ZAF	Norm	174	55.2	7.5	5.2	0	0	5.7	0	0	0	6.9	13.2	0	0	0	0	0	0	0	0	24.7
	Denny 2008	Y/N	C-S	29.4	18-54	C	PGMY9 /11		ASC	29	86.2	17.2	13.8	0	0	20.7	0	0	0	17.2	6.9	0	0	0	0	0	0	0	0	58.6
	Denny 2008	Y/N	C-S	28.3	18-54	C	PGMY9 /11		LSIL	137	91.2	21.2	13.9	0	0	22.6	0	0	0	21.9	16.8	0	0	0	0	0	0	0	0	67.2
	Denny 2008	Y/N	C-S	31.5	18-54	C	PGMY9 /11		HSIL	50	100	26	18	0	0	18	0	0	0	22	20	0	0	0	0	0	0	0	0	70
	Denny 2014b	Y/N	C-S	52	29-85	H	SPF	NLD	ICC	228	92.1	37.7	14.5	1.3	5.3	7.9	0.4	6.1	0.9	1.3	0	0.9	0	0.4	0.4	.	.	.	2.2	9.6
	Firnhaber 2009	Y	C-S	37	18-65	C	PGMY9 /11	ZAF	Norm	64	89.1	25	12.5	6.3	3.1	12.5	6.3	17.2	7.8	10.9	18.8	9.4	6.3	7.8	4.7	7.8	0	0	10.9	64.1
	Firnhaber 2009	Y	C-S	35	18-65	C	PGMY9 /11		HSIL+	23	100	60.9	8.7	13	13	43.5	13	17.4	26.1	21.7	13	26.1	17.4	17.4	26.1	8.7	0	0	60.9	100
	Firnhaber 2009	Y	C-S	36	18-65	C	PGMY9 /11		UNS	60	100	23.3	28.3	6.7	11.7	18.3	10	15	15	13.3	21.7	16.7	10	11.7	15	8.3	0	0	35	73.3
	Firnhaber 2010	Y	C-S	34	18-65	C	PGMY9 /11	ZAF	Norm	90	74.4	17.8	12.2	0	3.3	0	0	0	0	0	0	8.9	0	0	5.6	0	0	0	0	38.9
	Firnhaber 2010	Y	C-S	34	18-65	C	PGMY9 /11		ASC	12	100	8.3	8.3	0	0	0	0	0	0	0	0	8.3	0	0	8.3	0	0	0	0	50
	Firnhaber 2010	Y	C-S	34	18-65	C	PGMY9 /11		LSIL	51	98	31.4	35.3	0	9.8	0	0	0	0	0	0	19.6	0	0	19.6	0	0	0	0	68.6
	Firnhaber 2010	Y	C-S	34	18-65	C	PGMY9 /11		HSIL	34	94.1	44.1	2.9	0	14.7	0	0	0	0	0	0	23.5	0	0	17.6	0	0	0	0	61.8
	Firnhaber 2010	Y	C-S	34	18-65	C	PGMY9 /11		ICC	2	100	50	50	0	0	0	0	0	0	0	0	0	0	0	0	0	0	0	0	100
	Firnhaber 2010	Y	C-S	34	18-65	C	PGMY9 /11		UNS	2	100	50	50	0	0	0	0	0	0	0	0	0	0	0	0	0	0	0	0	100
	Jones 2007	N	C-S	.	>18	C	PGMY9 /11	ZAF	Norm	151	45	4	6.6	3.3	0.7	4.6	3.3	6.6	2.6	5.3	6	4	7.3	0	4	3.3	3.3	33.3	9.9	.
	Jones 2007	N	C-S	.	>18	C	PGMY9 /11		HSIL	6	100	16.7	33.3	16.7	33.3	33.3	16.7	33.3	16.7	0	0	0	33.3	50	16.7	16.7	0	0	50	.
	Jones 2007	N	C-S	.	>18	C	PGMY9 /11		UNS	24	45.8	8.3	0	4.2	4.2	4.2	4.2	8.3	8.3	12.5	8.3	4.2	4.2	8.3	4.2	4.2	0	0	12.5	.
	Kay 2003	N	C-S	35.3	19-65, 30-77	H	IH	ZAF	HSIL	129	88.4	56.6	14	10.9	14	2.3	0	0	0	3.9	0	0	3.1	0	0	0	0	2	7	12.4
	Kay 2003	N	C-S	54.6	19-65, 30-77	H	IH		ICC	50	94	82	10	2	10	2	0	0	0	0	0	0	2	2	0	0	0	0	0	16
	Marais 2000	N	C-S	37	21-50+	H	IH	ZAF	Norm	95	44.2	25.3	10.5	21.1	9.5	.	.	13.7	.	.	.	.	.	.	.	.	.	.	0	21.1
	Marais 2000	N	C-S	54	21-50+	H	IH		ICC	40	75	25	45	37.5	12.5	.	.	55	.	.	.	.	.	.	.	.	.	.	0	62.5
	Marais 2000	N	C-S	37	21-50+	H	IH		ASC+	95	71.6	44.2	20	33.7	11.6	.	.	27.4	.	.	.	.	.	.	.	.	.	.	0	36.8
	Marais 2008	Y/N	C-S	35	22-62	C	PGMY9 /11		Norm	29	93.1	17.2	6.9	3.4	13.8	17.2	3.4	0	10.3	0	3.4	17.2	13.8	3.4	0	3.4	3.4	6.5	17.2	0
	Marais 2008	Y/N	C-S	35	22-62	C	PGMY9 /11		LSIL	31	100	19.4	16.1	6.5	9.7	19.4	6.5	12.9	16.1	12.9	12.9	9.7	9.7	6.5	6.5	9.7	6.5	0	25.8	0
	Marais 2008	Y/N	C-S	35	22-62	C	PGMY9 /11		HSIL	55	92.7	36.4	16.4	12.7	10.9	12.7	5.5	10.9	3.6	10.9	3.6	3.6	10.9	1.8	9.1	5.5	3.6	0	12.7	0

Country (S) N [%] {Region} GNI	Study	HIV (Y/N)	Design	Median Age	Age range	Pathology	Primer	HPV genotyping location	Cervical Lesion/Norm	N	Any	HPV-specific prevalence (% of all cases tested)																	Other	Multi
												16	18	31	33	35	39	45	51	52	53	56	58	59	66	68	6	11		
	McDonald 2012	N	C-S	.	17-65	C	PGMY9/11		Norm	1261	71.5	11.6	6.7	5.7	5.3	12	4	8.4	5.9	7.1	.	4.4	9.1	5.2	.	6.7	.	.	0	.
	McDonald 2012	N	C-S	.	17-65	C	PGMY9/11		LSIL	198	86.9	11.6	11.1	8.6	7.6	20.7	3.5	9.6	11.6	10.6	.	7.6	7.1	7.1	.	8.1	.	.	0	.
	McDonald 2012	N	C-S	.	17-65	C	PGMY9/11		HSIL	183	90.2	27.3	9.3	9.3	11.5	23.5	1.6	10.4	3.3	8.2	.	2.2	12	1.6	.	3.3	.	.	0	23.5
	Moodley 2009	Y	C-S	31	20-60	C	PGMY9/11	ZAF	HSIL	10	100	30	20	20	20	30	30	40	30	0	0	0	30	0	20	0	0	0	0	90
	Ramesar 1996	N	C-S	38	19-85	C	IH	N.S.	Norm	192	13	1	3.6	0	0	0	.	.	.	.	.	.	.	.	.	.	.	.	6.3	0.5
	Ramesar 1996	N	C-S	38	19-85	C	IH		ASC	9	22.2	11.1	11.1	0	0	0	.	.	.	.	.	.	.	.	.	.	.	.	0	0
	Ramesar 1996	N	C-S	38	19-85	C	IH		LSIL	5	40	0	0	0	20	0	.	.	.	.	.	.	.	.	.	.	.	.	20	0
	Richter 2008	Y	C-S	32	22-64	C	PGMY9/11	ZAF	Norm	16	93.8	6.3	0	6.3	18.8	31.3	6.3	12.5	12.5	0	6.3	6.3	12.5	0	6.3	18.8	12.5	0	18.8	87.5
	Richter 2008	Y	C-S	32	22-64	C	PGMY9/11		ASC	7	100	42.9	0	0	14.3	14.3	14.3	28.6	0	28.6	28.6	0	28.6	0	0	0	28.6	0	28.6	100
	Richter 2008	Y	C-S	32	22-64	C	PGMY9/11		LSIL	3	100	33.3	0	33.3	0	33.3	33.3	0	0	0	0	66.7	0	0	0	0	0	0	0	100
	Richter 2008	Y	C-S	32	22-64	C	PGMY9/11		HSIL	4	100	0	25	0	50	25	0	25	0	0	25	25	25	0	0	0	0	0	25	75
	Said 2009	N	C-S	.	.	C	PGMY9/11	ZAF	Norm	99	40.4	8.1	5.1	2	0	2	7.1	6.1	5.1	3	0	0	5.1	3	0	1	0	0	13.1	19.2
	Said 2009	N	C-S	.	.	C	PGMY9/11		ASC	23	100	17.4	17.4	17.4	0	8.7	8.7	8.7	8.7	8.7	0	8.7	4.3	8.7	13	4.3	13	0	30.4	47.8
	Said 2009	N	C-S	.	.	C	PGMY9/11		LSIL	16	100	25	25	0	6.3	31.3	6.3	6.3	0	6.3	0	0	18.8	12.5	18.8	18.8	0	0	81.3	43.8
	Said 2009	N	C-S	.	.	C	PGMY9/11		HSIL	8	87.5	12.5	12.5	0	0	50	0	0	0	0	0	0	25	12.5	25	0	0	0	0	37.5
	Said 2009	N	C-S	.	.	C	MY9/11		HSIL+	7	100	0	14.3	0	0	14.3	0	0	14.3	0	0	0	42.9	0	0	28.6	0	0	0	42.9
	Williamson 1989	N	C-S	.		C	IH	ZAF	HSIL	98	67.3	16.3	1	2	8.2	0	.	.	.	.	.	.	.	.	.	.	0	0	35.7	2
	Williamson 1994	N	C-S	49	28-77	H	IH	ZAF	ICC	68	80.9	45.6	1.5	5.9	5.9	0	.	1.5	.	.	.	.	.	.	.	.	0	0	25	5.9
**Sudan** (1) 85 [0.3] {Eastern Africa} Low Middle Income	Abate 2012b	N	C-S	45	21-85	H	SPF	SDN	HSIL	1	100	100	0	0	0	0	0	0	0	0	0	0	0	0	0	0	0	0	0	0
	Abate 2012b	N	C-S	45	21-85	H	SPF		ICC	84	94	77.4	19	6	11.9	11.9	3.6	16.7	0	14.3	6	0	15.5	1.2	0	0	9.5	0	1.2	4.8
**Tanzania** (4) 4447 [16.7] {Eastern Africa} Low Income	Bosch 1995f	N	C-S	34	.	H	PGMY9/11	USA	ICC	49	87.8	44.9	24.5	0	.	.	0	10.2	.	.	.	.	.	0	.	.	0	0	0	.
	Dartell 2012	Y/N	C-S	38	15-82	C	SPF	DNK	Norm	3235	14.8	2.1	1.2	0.9	0.7	1.9	0.6	0.7	2.1	3.2	0.6	0.9	0.6	0.1	1.6	1.1	0	0	0	4.4
	Dartell 2012	Y/N	C-S	38	15-82	C	SPF		ASC	169	41.4	9.5	4.7	3.6	2.4	4.7	0.6	0.6	6.5	8.9	1.8	1.2	3	0	4.1	0.6	0	0	0	13
	Dartell 2012	Y/N	C-S	38	15-82	C	SPF		LSIL	62	77.4	9.7	4.8	4.8	3.2	12.9	6.5	3.2	12.9	14.5	6.5	9.7	8.1	1.6	6.5	4.8	0	0	0	30.6
	Dartell 2012	Y/N	C-S	38	15-82	C	SPF		HSIL	132	93.9	34.1	18.9	3	9.8	13.6	2.3	3.8	7.6	25	4.5	3	5.3	0	10.6	2.3	0	0	4.5	34.8
	Dartell 2012	Y/N	C-S	38	15-82	C	SPF		ICC	5	100	80	20	0	0	0	0	0	0	0	0	0	0	0	0	0	0	0	0	0

Country (S) N [%] {Region} GNI	Study	HIV (Y/N)	Design	Median Age	Age range	Pathology	Primer	HPV genotyping location	Cervical Lesion/Norm	N	Any	HPV-specific prevalence (% of all cases tested)																	
												16	18	31	33	35	39	45	51	52	53	56	58	59	66	68	6	11	Other	Multi
	Mayaud 2003	N	C-S	23.4	15 to 44	C	MY9/11	GBR	Norm	542	31.5	5.7	4.1	1.1	3.1	0.2	0	0	1.1	0.4	0	0	0	0	0	0	0	0	11.1	8.5
	Mayaud 2003	N	C-S	23.4	15 to 44	C	MY9/11		LSIL	10	100	30	10	20	20	10	0	0	0	10	0	0	0	10	0	0	0	0	30	20
	Mayaud 2003	N	C-S	23.4	15 to 44	C	MY9/11		HSIL	9	100	11.1	11.1	11.1	22.2	11.1	0	0	11.1	0	0	0	0	0	0	0	0	0	44.4	22.2
	Vidal 2011	N	C-S	40.3	.	C	PGMY9/11	USA	Norm	148	13.5	2.7	2	0.7	0	0.7	0	2	1.4	0	0.7	0.7	4.1	0.7	2	0.7	0	0	7.4	13.5
	Vidal 2011	N	C-S	35.7	.	C	PGMY9/11		LSIL	21	57.1	9.5	0	0	0	19	4.8	14.3	14.3	4.8	14.3	0	23.8	14.3	0	0	0	5.9	42.9	100
	Vidal 2011	N	C-S	44.7	.	C	PGMY9/11		HSIL	17	82.4	17.6	17.6	11.8	11.8	11.8	0	5.9	5.9	11.8	29.4	0	5.9	0	11.8	5.9	5.9	0	147.1	76.5
	Vidal 2011	N	C-S	55.2	.	C	PGMY9/11		ICC	48	68.8	60.4	14.6	12.5	6.3	27.1	0	22.9	0	10.4	0	0	4.2	0	0	0	22.9	15.8	16.7	58.3
**Uganda** (5) 959 [3.6] {Eastern Africa} Low Income	Blossom 2007	Y	C-S	26	18-51	C	PGMY9/11	USA	HSIL	19	73.7	26.3	5.3	5.3	5.3	5.3	0	10.5	10.5	21.1	5.3	15.8	21.1	0	10.5	5.3	10.5	2.3	0	52.6
	Bosch 1995e	N	C-S	34	.	C	PGMY9/11	USA	ICC	43	93	53.5	16.3	0	.	.	0	4.7	.	.	.	.	.	0	.	.	0	0	0	.
	Odida 2008	N	C-S	.	.	C	SPF	NLD	ICC	181	63	29.8	18.2	2.2	0	1.1	1.1	7.2	1.7	1.1	0	0	0	0	0	0	0	0	2.8	2.2
	Odida 2010	N	C-S	.		C	SPF	NLD	ICC	171	90.1	42.7	17.5	0.6	1.8	3.5	1.2	8.8	1.8	1.2	0	0	0.6	0	3.5	.	0	0	3.5	6.4
	Odida 2011	Y/N	C-S	41	18-74	C	SPF	NLD	Norm	309	30.7	3.2	2.6	1	1.6	2.3	0.3	0	2.6	4.2	1.3	1.3	1	0.6	1.6	.	1	0.8	12.9	6.5
	Odida 2011	Y/N	C-S	47	18-74	C	SPF		ICC	236	89.8	47	20.8	1.3	1.7	4.2	1.3	9.3	1.7	2.5	0	0.8	0.8	0.4	0	0	0.8	0	6.4	8.1
**Zaire** (DRC)(1) 10 [0.0] {Middle Africa} Low Income	Icenogle 1992	Y	C-S	.		C	GP5/6	USA	LSIL	8	75	12.5	12.5	12.5	12.5	0	.	.	.	.	.	.	.	.	.	.	.	.	25	62.5
	Icenogle 1992	Y	C-S	.		C	MY9/11		HSIL	2	100	100	0	0	0	0	.	.	.	.	.	.	.	.	.	.	.	.	0	50
**Zambia** (2) 218 [0.8] {Eastern Africa} Low Income	Ng'andwe 2007	Y/N	C-S	22	15-38	C/H	MY9/11	USA	Norm	70	65.7	15.7	15.7	0	2.9	2.9	0	1.4	1.4	0	0	0	2.9	0	.	0	5.7	0	7.1	0
	Sahasrabuddhe 2007	Y	C-S	36.2	23-49	C	PGMY9/11	USA	Norm	10	80	0	20	0	0	20	10	0	0	20	10	10	10	0	0	10	0	3.8	130	30
	Sahasrabuddhe 2007	Y	C-S	36.2	23-49	C	PGMY9/11		ASC	26	96.2	23.1	11.5	7.7	0	15.4	11.5	11.5	7.7	30.8	23.1	3.8	7.7	3.8	7.7	11.5	0	0	176.9	65.4
	Sahasrabuddhe 2007	Y	C-S	36.2	23-49	C	PGMY9/11		LSIL	35	97.1	5.7	11.4	8.6	11.4	17.1	11.4	17.1	14.3	40	17.1	17.1	20	17.1	28.6	14.3	8.6	0	234.3	91.4
	Sahasrabuddhe 2007	Y	C-S	36.2	23-49	C	PGMY9/11		HSIL	49	100	14.3	10.2	20.4	8.2	12.2	16.3	24.5	18.4	38.8	20.4	14.3	30.6	0	12.2	14.3	0	0	12.2	100
	Sahasrabuddhe 2007	Y	C-S	36.2	23-49	C	PGMY9/11		ICC	28	100	35.7	17.9	21.4	14.3	28.6	10.7	17.9	21.4	46.4	25	14.3	35.7	14.3	10.7	14.3	0	0	14.3	78.6
**Zimbambwe** (3) 564 [2.1] {Eastern Africa} Low Income	Gravitt 2002	N	C-C	32	25-55	C	MY9/11	USA	Norm	213	30	4.7	4.7	0.5	3.3	0.5	1.9	1.4	5.2	6.6	4.2	3.3	3.8	0	2.8	1.9	2.3	1	7.5	16
	Gravitt 2002	N	C-C	32	25-55	C	MY9/11		HSIL	210	77.1	30.5	20.5	12.9	14.8	10	5.2	5.7	11.4	19	16.2	8.6	21.9	5.2	6.7	6.7	6.2	14	32.4	52.4
	Stanczuk 2003a	N	C-S	44	24-70	H	IH	ZAF	ICC	43	97.7	58.1	14	2.3	27.9	0	0	0	0	0	0	0	2.3	0	.	.	.	.	0	14
	Stanczuk 2003b	N	C-S	46	22-77	H	IH		ICC	98	100	62.2	19.4	4.1	39.8	1	0	0	0	0	0	0	1	0	0	0	0	0	0	25.5
**Mixed 4** (1) 155 [0.7] {Eastern and	Dols 2012	Y	C-S	.	>18	C	SPF		Norm	138	42	13	4.3	3.6	8	4.3	2.2	0.7	7.2	12.3	0	2.2	2.2	0	5.1	1.4	.	.	0	.
	Dols 2012	Y	C-S	.	>18	C	SPF		HSIL	17	94.1	29.4	11.8	0	5.9	23.5	11.8	0	11.8	41.2	0	0	0	0	11.8	11.8	.	.	0	.

											HPV-specific prevalence (% of all cases tested)																	
Country (S) N [%] {Region} GNI	**Study**	**HIV (Y/N)**	**Design**	**Median Age**	**Age range**	**Pathology**	**Primer**	**HPV genotyping location**	**Cervical Lesion/Norm**	**N**	**Any**	**16**	**18**	**31**	**33**	**35**	**39**	**45**	**51**	**52**	**53**	**56**	**58**	**59**	**66**	**68**	**6**	**11**	**Other**	**Multi**
Southern Africa} Mixed Economies	Dols 2012	Y	C-S	.	>18	C	SPF		UNS	39	89.7	38.5	2.6	5.1	7.7	7.7	2.6	0	7.7	23.1	0	7.7	20.5	2.6	33.3	5.1	.	.	0	.
**Mixed 5** (1) 468 [0.9] {Eastern and Southern Africa} Mixed Economies	De Vuyst 2011	Y	C-C	43.2		H	GP5/6		ICC	235	95.7	50.2	15.7	1.3	2.6	7.2	0.9	14	0.4	1.7	0	2.1	3	0.4	0	2.1	0	0	0.9	11.1

Abbreviations Used:
Design: C-S: Cross sectional; C-C: case control
Pathology reading: C- Cytology; H -Histology
Primer: PYGMY9/11; GP5/6; MY9/11; SPF10; IH: in house primer;
Location of HPV genotype: USA: United States of America; NLD: Netherlands; ITA: Italy; ZAF: South Africa; FRA: France; ESP: Spain; ETH: Ethiopia; GBR: United Kingdom; SWE: Sweden; BEL: Belgium; JPN: Japan; N.S.: Not stated; MAR: Morocco; SDN: Sudan; DNK: Denmark;
Cervical Lesion / Norm: ICC: invasive cervical carcinoma; ASC: atypical squamous cells of undetermined significance; LSIL: low-grade squamous intraepithelial lesion; HSIL: high-grade squamous intraepithelial lesion; Norm: Normal; ASC+: women who reported ASCUS or higher lesions without specifying the exact lesion type; HSIL+: women who reported HISL or higher lesions without specifying whether it is HSIL or ICC; UNS**:** Unspecified low lesion type.
